# Loss of NDRG2 enhanced activation of the NF-κB pathway by PTEN and NIK phosphorylation for ATL and other cancer development

**DOI:** 10.1038/srep12841

**Published:** 2015-08-13

**Authors:** Tomonaga Ichikawa, Shingo Nakahata, Masahiro Fujii, Hidekatsu Iha, Kazuhiro Morishita

**Affiliations:** 1 Division of Tumor and Cellular Biochemistry, Department of Medical Sciences, University of Miyazaki, 5200 Kihara, Kiyotake, Miyazaki 889-1692, Japan; 2 Division of Virology, Graduate School of Medical and Dental Sciences, Niigata University, Niigata 951-8510, Japan; 3 Department of Microbiology, Faculty of Medicine, Oita University, Yufu 879-5593, Oita, Japan

## Abstract

The activation of nuclear factor kappa B (NF-κB) signaling has a central role in the development of adult T-cell leukemia/lymphoma (ATL) and many other cancers. However, the activation mechanism of the NF-κB pathways remains poorly understood. Recently, we reported that N-myc downstream-regulated gene 2 (NDRG2) is a negative regulator of the phosphoinositide 3-kinase (PI3K)/AKT pathway by promoting the active dephosphorylated form of PTEN at its C-terminus via the recruitment of PP2A. Additionally, the down-regulation of NDRG2 expression promotes the inactive phosphorylated form of PTEN, which results in constitutively active PI3K/AKT signaling in various cancer cell types. Here, we investigated the involvement of NDRG2 in modulating NF-κB signaling. The forced expression of NDRG2 in ATL cells down-regulates not only the canonical pathway by inhibiting AKT signaling but also the non-canonical pathway by inducing NF-κB-inducing kinase (NIK) dephosphorylation via the recruitment of PP2A. Therefore, NDRG2 works as a PP2A recruiter to suppress not only PI3K/AKT signaling but also NF-κB signaling, which is particularly important in host defenses or immune responses to Human T-cell leukemia virus type 1 (HTLV-1) infection. Furthermore, the loss of NDRG2 expression might play an important role in the progression of tumor development after HTLV-1 infection.

Human T-cell leukemia virus type 1 (HTLV-1) is an oncogenic retrovirus associated with an aggressive form of CD4+ T-cell leukemia termed adult T-cell leukemia/lymphoma (ATL). Although the molecular mechanism of leukemogenesis has not yet been completely elucidated, aberrations in signal transduction in HTLV-1-infected T cells play an important role in the development of the disease. Among them, nuclear factor kappa B (NF-κB) is constitutively activated in HTLV-1-infected T and ATL cells, and activation of the NF-κB signaling pathway is dependent or independent of HTLV-1 Tax expression along with many molecular events. Moreover, several studies have suggested that NF-κB plays an essential role in the pathogenesis of ATL and is one of important molecular targets for the prevention and treatment of ATL^1^,^2^,^3^,^4^,^5^.

NF-κB comprises a family of transcription factors that play critical roles in inflammation, immunity, cell proliferation, differentiation, survival and cancer development^6^,^7^. NF-κB signaling is activated through canonical and non-canonical pathways, and the activation of the canonical pathway is mainly triggered by infection and cytokine stimuli such as lipopolysaccharide (LPS), tumor necrosis factor-α (TNFα) and interleukin-1β. Moreover, the activation of phosphoinositide 3-kinase (PI3K) and its downstream kinase AKT, one of the major oncogenic pathways, modulates the activation and phosphorylation of IKKs in the canonical pathway^8^,^9^,^10^,^11^,^12^. The activation of NF-κB by Tax is mediated by the direct binding of Tax to the regulatory subunit of I-κB kinase (IKK) NF-κB essential modulator (NEMO), which is also known as IKKγ. This interaction results in the constitutive activation of IKKα and IKKβ, the degradation of all I-κBs, and the activation of both the canonical and non-canonical NF-κB pathways. On the other hand, as a Tax-independent molecular mechanism of NF-κB activation, in canonical pathway, p47, a novel binding partner of polyubiquitinated NEMO, triggers the lysosomal degradation of NEMO and the down-regulation of p47 expression in ATL cells, thereby resulting in enhanced TNF-α- or IL-1-induced IKK activation through increased degradation of NEMO^13^. In the non-canonical pathway, high expression of NF-κB inducible kinase (NIK) was found in ATL through low expression of miR31^14^ and the accumulation of NIK protein led to the activation of IKK followed by the phosphorylation of p100 and processing to p52, resulting in the nuclear translocation of p52/RelB heterodimers.

Recently, we identified N-myc downstream-regulated gene 2 (NDRG2) as a novel PTEN-binding protein that recruits protein phosphatase 2A (PP2A) to regulate the dephosphorylation of PTEN at Serine380, Threonine382 and Threonine383 in the C-terminal domain of PTEN. NDRG2 expression was down-regulated in ATL via genetic deletion and DNA promoter methylation, resulting in the activation of the PI3K-AKT pathway through increased PTEN phosphorylation at its C-terminus, representing its inactive form^15^. Moreover, NDRG2 is reported to negatively regulate several signal transduction pathways such as MAPK^16^,^17^, JAK-STAT^18^,^19^ and NF-κB^20^,^21^, although the real function of NDRG2 is still unknown. Because NDRG2 acts to recruit PP2A to PTEN, NDRG2 might play a pivotal role in attenuating the phosphorylation of important signaling molecules in each of the signal transduction pathways by recruiting PP2A.

Therefore, we investigated whether NDRG2 expression could modulate NF-κB signaling in ATL and other solid cancers. In this manuscript, the forced expression of NDRG2 remarkably inhibited the canonical NF-κB pathway by inhibiting AKT signaling in ATL and solid cancer cells. In the non-canonical pathway, NDRG2 expression dephosphorylates NIK, which is overexpressed and highly phosphorylated (active) in ATL, through the recruitment of PP2A. Therefore, NDRG2 works as a recruiter of PP2A to suppress the important signaling pathways for defending against infection or immune responses, and the loss of NDRG2 expression might play an important role in the progression of tumor development.

## Results

### Suppression of the inflammatory-induced canonical NF-κB pathway through NDRG2 overexpression in OSCC

**Figure 1:**
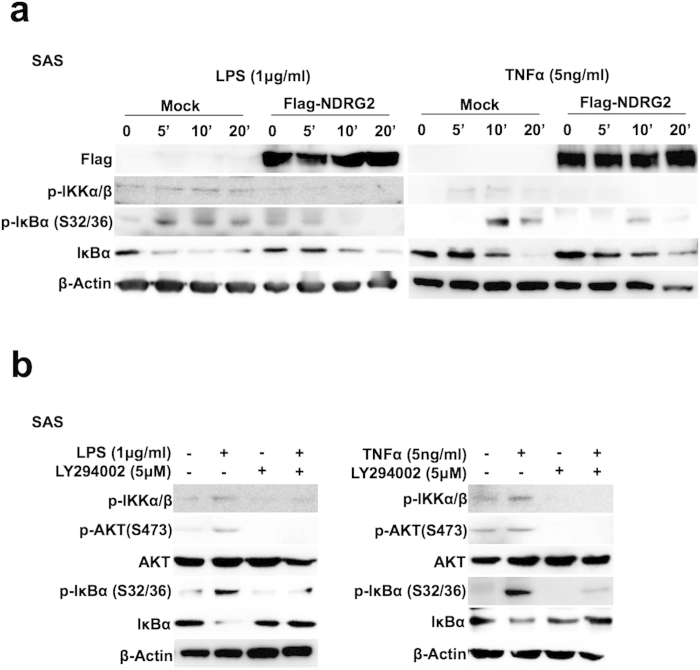
Suppression of the inflammation-induced canonical NF-κB pathway through the overexpression of NDRG2 in OSCC cells upon stimulation. (**a**) Stably transfected SAS cell lines were cultured in serum-free DMEM for 24 h. The quiescent cells were treated with LPS (1 μg/ml) and TNFα (5 ng/ml) in serum-free medium and were then subjected to Western bolt analysis as indicated. The results are representative of three independent experiments. (**b**) Cells were pretreated with or without LY294002 (5 μM) in serum-free DMEM and subjected to treatment with LPS or TNFα, followed by western blot analysis, as indicated. The results are representative of three independent experiments.

We recently showed that NDRG2 regulates the phosphorylation status of PTEN at its C-terminus by recruiting PP2A and suppresses PI3K/AKT signaling by dephosphorylating PTEN, the active form of PIP3 phosphatase^15^. NDRG2 has been reported to suppress tumor progression through the regulation of NF-κB signaling^20^,^21^; however, the detailed molecular mechanism by which NDRG2 regulates the canonical and/or non-canonical NF-κB pathways remains unclear. Because the loss of NDRG2 expression was found in the majority of OSCC^22^, we examined the effects of NDRG2 on the stimuli-induced canonical and/or non-canonical NF-κB pathways in cancer cells by establishing stable NDRG2-expressing (NDRG2) or mock clone (Mock) oral squamous cell carcinoma (OSCC) cell lines (SAS and HSC3) after transfecting with an NDRG2-expressing plasmid or a Mock vector, as a control (Fig. 1a and supplementary Fig. S1a). After LPS or TNFα stimulation in these SAS and HSC3 clones, IKKα/β and IκBα-Ser32/36 phosphorylation coupled with IκBα degradation were observed in mock cells; however, IKK and IκBα-Ser32/36 phosphorylation and IκBα degradation were significantly suppressed in the NDRG2 cell lines (Fig. 1a and supplementary Fig. S1a–c). In contrast, because the expression of p100 and processing of p52 in the non-canonical pathway were not detected in most of the OSCC cell lines (supplementary Fig. S1d), the canonical NF-κB pathway was likely preferentially active in OSCC cells. Moreover, the p65 transcription factor in the canonical pathway was translocated to the nucleus of Mock cells after treatment with LPS or TNFα; however, the nuclear translocation of p65 was inhibited in the NDRG2/SAS cell lines (supplementary Fig. S1e). In addition, the transcriptional activity of NF-κB was increased following LPS or TNFα treatment in Mock cells; however, the NF-κB transcriptional activity was significantly reduced in NDRG2/SAS cells (supplementary Fig. S1f). Therefore, NDRG2 expression in OSCC might mainly suppress the activity of the canonical NF-κB pathway.

Because NDRG2 suppressed PI3K/AKT signaling through PTEN phosphorylation^15^ and the activation PI3K/AKT signaling pathway might enhance the activation of the canonical NF-κB activation by cytokine stimulation^8^,^9^, the suppressive activity of the canonical NF-κB pathway by NDRG2 expression might be due to the suppression of PI3K/AKT signaling. Therefore, to gain insight into the suppressive mechanism of the inflammation-induced canonical NF-κB pathway through NDRG2 expression, we determined the activity of the NF-κB pathway after treating these cell lines with a PI3K inhibitor. After stimulation with LPS or TNFα, the phosphorylation status of PTEN-Ser380/Thr382/Thr383, PDK1-Ser241, AKT-Thr308, AKT-Ser473, GSK3β-Ser9 and S6-Ser240/244 and the expression of inflammatory target genes downstream of NF-κB signaling (MCP-1, ICAM-1, iNOS, COX-2, MMP-2, MMP-9 and IL6) were increased in the mock cells; however, the phosphorylation levels of these target proteins and the expression levels of the inflammatory genes were completely suppressed in the NDRG2/SAS cell line, even after stimulation with LPS or TNFα (supplementary Fig. S1g and h). Therefore, after control OSCC (SAS) cells were treated with each NF-κB stimulator in the presence or absence of the PI3K inhibitor LY294002, the NF-κB signaling activity was assessed. Treatment with LY294002 inhibited the LPS- or TNFα-induced phosphorylation of IKKα/β, IκBα-Ser32/36, and AKT-Ser473, and the degradation of IκBα (Fig. 1b). Moreover, the enhanced NF-κB transcriptional activity after LPS or TNFα stimulation was also significantly inhibited by treatment with the PI3K inhibitor (supplementary Fig. S1i).

**Figure 2:**
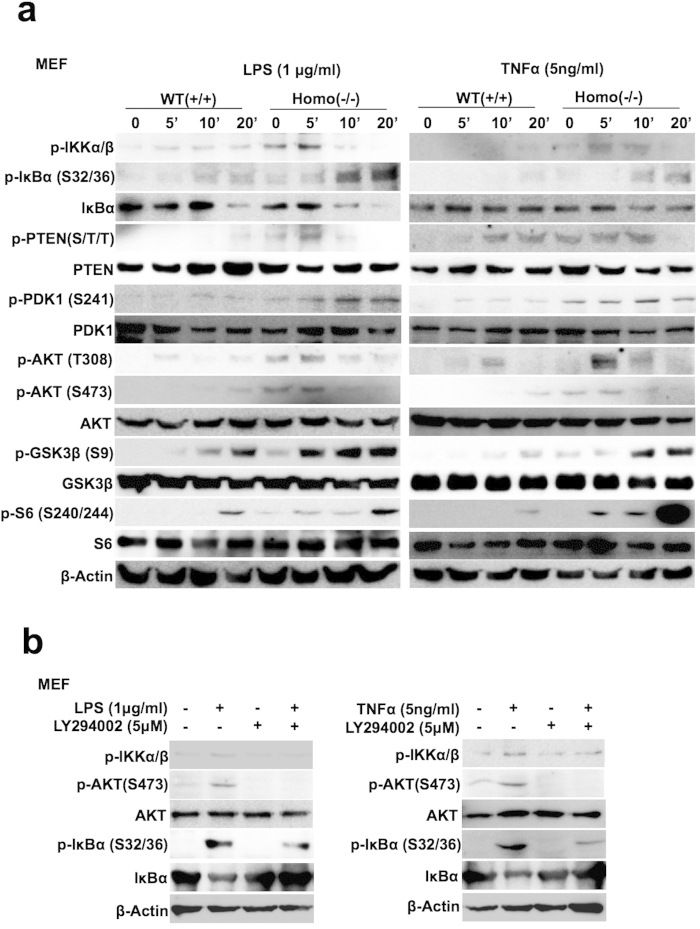
Suppression of the inflammatory-induced canonical NF-κB pathway in Ndrg2-deficient MEF cells upon stimulation. (**a**) Wild-type (+/+) and Ndrg2-deficient (–/–) mouse embryonic fibroblast (MEF) cells were cultured with serum-free DMEM for 24 h. The quiescent cells were treated with LPS (1 μg/ml) and TNFα (5 ng/ml) in serum-free medium and then subjected to Western bolt analysis, as indicated. The results are representative of three independent experiments. (**b**) MEFs were pretreated with or without LY294002 (5 μM) in serum-free DMEM and subjected to treatment with LPS or TNFα, followed by western blot analysis, as indicated. The results are representative of three independent experiments.

To confirm the results, we established mouse embryonic fibroblasts (MEFs) from Ndrg2-deficient and wild-type mice, and these MEF cell lines were analyzed for the activation of PI3K/AKT and NF-κB signaling stimulated by LPS or TNFα. As shown in Fig. 2a, the phosphorylation status of PI3K/AKT signaling molecules (PTEN-Ser380/Thr382/Thr383, PDK1-Ser241, AKT-Thr308, AKT-Ser473, GSK3β-Ser9 and S6-Ser240/244) were enhanced and prolonged in Ndrg2-deficient MEF cells; the canonical NF-κB signaling pathway (phosphorylation of IKK and IκBα-Ser32/36 and degradation of IκBα) was also enhanced in the Ndrg2-deficient MEF cells (Fig. 2a and supplementary Fig. S2a). The NF-κB transcriptional activity following LPS or TNFα treatment was enhanced in Ndrg2-deficient MEF cells (supplementary Fig. S2b). Similar to OSCC cell lines, expression of p100 and processed p52 in non-canonical pathway were not detected in wild-type and Ndrg2-deficient MEF cells (supplementary Fig. S2c). The induction of phosphorylated IKKα/β, IκBα (Ser32/36) and the degradation of IκBα following LPS or TNFα stimulation in MEFs were inhibited by treatment with LY294002, which suppresses phosphorylated AKT (Ser473) (Fig. 2b). Furthermore, co-treatment with LY294002 and LPS or TNFα suppressed the transcriptional activity of NF-κB compared with treatment with LPS or TNFα alone (supplementary Fig. S2d). These finding suggest that NDRG2 may suppress the canonical NF-κB pathway through inhibiting AKT activity.

### Regulation of the canonical and non-canonical NF-κB pathways by NDRG2 in HTLV-1+ and ATL cells

**Figure 3:**
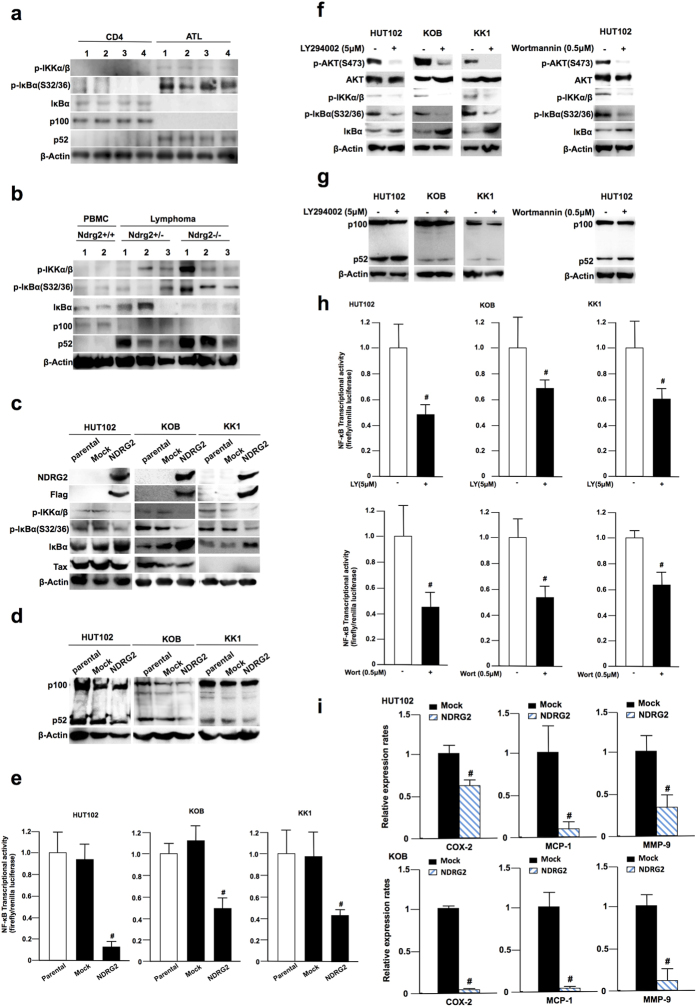
Regulation of the canonical and non-canonical NF-κB pathways by NDRG2 in HTLV-1+ and ATL cells. (**a**) Normal CD4+ lymphocytes and primary ATL samples were subjected to western blot analysis of the canonical and non-canonical NF-κB pathways. (**b**) PBMC from wild-type mice and lymphomas from Ndrg2-deficient mice were subjected to western blot analysis of the canonical and non-canonical NF-κB pathways. (**c**) Stably transfected HUT102, KOB and KK1 cells were subjected to western blot analysis of the canonical NF-κB pathway. Note that the amount of HTLV-1 Tax protein in the three cell lines was found to differ. The results are representative of three independent experiments. (**d**) Stably transfected HUT102, KOB and KK1 cells were subjected to western blot analysis of the non-canonical NF-κB pathway. The results are representative of three independent experiments. (**e**) Cells were transfected with pNF-κB-Luc and pRL-TK plasmids and then subjected to NF-κB reporter assays. The data are expressed as the mean ± s.d. Student’s t-test was used for the statistical analysis (p < 0.05). (**f** and **g**) Cells were pretreated with or without LY294002 (5 μM) or Wortmannin (0.5 μM) and incubated with 10% FBS DMEM for another 24 h, followed by western blot analysis, as indicated. The results are representative of three independent experiments. (**h**) Cells were transfected with pNF-κB-Luc and pRL-TK plasmids, pretreated with or without LY294002 (5 μM) or Wortmannin (0.5 μM), incubated with 10% FBS DMEM for another 24 h, and then subjected to NF-κB reporter assays. The data are expressed as the mean ± standard deviation (s.d). Student’s t-test was used for the statistical analysis (p < 0.05). (**i**) Quantitative real-time RT-PCR analysis of inflammatory targets mRNA in ATL-Mock and –NDRG2 cells. The data are expressed as the mean ± s.d of triplicate wells. Student’s t-test was used for statistical analysis (p < 0.05).

Although non-canonical NF-κB signaling was not activated in OSCC and MEF cells, the non-canonical NF-κB pathway was highly activated by the HTLV-1-encoded oncoprotein Tax and/or NIK overexpression in HTLV-1-infected ATL cells (HTLV-1+ cells)^23^,^24^,^25^,^26^,^27^,^28^. We analyzed activation status of NF-κB signaling pathway in primary ATL cells from acute-type ATL patients and CD4+ lymphocytes from healthy volunteers as controls (Fig. 3a). In activation status of the canonical NF-κB pathway, levels of the phosphorylated IKK-Ser176/180 and IκBα-Ser32/36 were remarkably higher and the protein level of IκBα was significantly lower than those in control CD4+ lymphocytes. In addition, the p100 processing to p52 in non-canonical pathway was increased in the majority of ATL samples, indicating that both of the canonical and non-canonical NF-κB signaling pathways were constitutively activated in ATL cells. Because Ndrg2-deficient mice developed various types of tumors including T cell lymphoma^15^, we also analyzed the activation status of the NF-κB signaling pathway in Ndrg2-deficient lymphoma cells (Fig. 3b). The western blots demonstrated that the canonical NF-κB signaling was constantly activated with increased levels of the phosphorylated IKK-Ser176/180 and IκBα-Ser32/36 proteins, and with reduced protein levels of IκBα in lymphoma in Ndrg2-deficient (Hetero and Homo) as compared with lymphocytes in the peripheral blood of wild-type mice. Furthermore, p100 processing to p52 was evident in most Ndrg2-deficient lymphoma cells, suggesting that the loss of NDRG2 involves the activation of the canonical and non-canonical NF-κB signaling pathway *in vivo*. Therefore, to explore whether NDRG2 regulates the canonical and non-canonical NF-κB pathways in ATL cell lines, we assessed the activation status of the canonical and non-canonical NF-κB pathways in three HTLV-1+ cell lines with various levels of HTLV-1 Tax expression (high expression in HUT102, middle in KOB and low in KK1) and whether NDRG2-introduction to these cell lines might affect the activation status of NF-κB signaling, including the NF-κB transcriptional activity. The canonical pathway was activated in all three HTLV-1+ cell lines, with high phosphorylation levels of IKK-Ser176/180 and IκBα-Ser32/36 that were not dependent on the expression level of Tax; additionally, the suppression of IKK phosphorylation and the accumulation of IκBα protein were detected in all NDRG2+ cell lines (Fig. 3c and supplementary Fig. S3a). Furthermore, bands representing p52 processed from p100 in the non-canonical pathway were observed in all three cell lines, and the level of processing of p100 to p52 was suppressed in the NDRG2+ cell lines (Fig. 3d and supplementary Fig. S3b), suggesting that the canonical and non-canonical pathways were activated in HTLV-1+ cell lines and that the transcriptional activities of both pathways were suppressed by NDRG2 expression (Fig. 3e).

In the next experiment to investigate whether the activation of the non-canonical pathway is regulated by PI3K/AKT signaling, we measured the activity of the non-canonical pathway after the treatment of the three HTLV-1+ cell lines with the PI3K inhibitor LY294002 and Wortmannin. As shown in Fig. 3f and supplementary Fig. S3c and d, treatment with the PI3K inhibitor inhibited the phosphorylation of IKK-Ser176/180, IκBα-Ser32/36 and AKT and also suppressed the protein degradation of IκBα in the canonical pathway. However, the level of p100 processed to p52 in the non-canonical pathway following PI3K inhibitor treatment was the same as that without PI3K inhibitor treatment in the three HTLV-1+ cell lines (Fig. 3g and supplementary Fig. S3e). Moreover, we measured the NF-κB transcriptional activity following the PI3K inhibitor treatment. Although the NF-κB transcriptional activity was reduced to approximately 20 to 40% of the control activity after the forced expression of NDRG2, the NF-κB transcriptional activity was only reduced to approximately 50 to 60% of the control activity by the PI3K inhibitor treatment (Fig. 3h). Furthermore, the levels of NF-κB-regulated inflammatory target genes were significantly decreased in NDRG2+ cell lines compared with mock cells (Fig. 3i). Because the reduced rate of NF-κB transcriptional activity following treatment with the PI3K inhibitor did not match the rate of NF-κB transcriptional activity after NDRG2 overexpression, NDRG2 might have a function in either indirectly or directly regulating the activity of the non-canonical pathway in addition to its function in PI3K/AKT inhibition.

### Suppression of the non-canonical NF-κB pathway through the dephosphorylation of NIK by the NDRG2/PP2A complex

**Figure 4:**
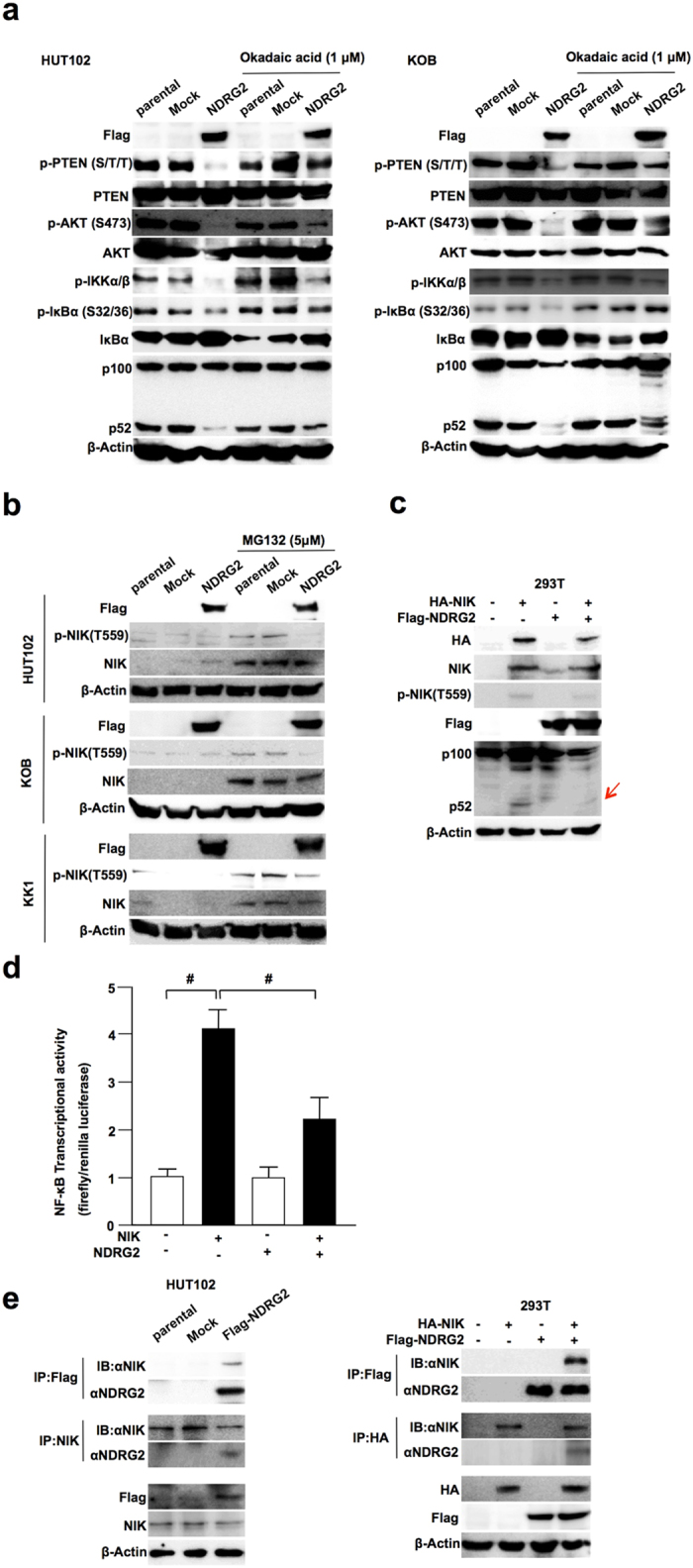
Regulation of the non-canonical NF-κB pathway via the interaction with NIK and NDRG2. (**a**) Stably transfected HUT102, KOB and KK1 cells were pretreated with or without Okadaic acid (1 μM) and incubated with 10% FBS DMEM for another 24 h, followed by western blot analysis, as indicated. The results are representative of three independent experiments. (**b**) Stably transfected HUT102, KOB and KK1 cells were pretreated with or without MG132 (5 μM) and incubated with 10% FBS DMEM for another 24 h, followed by western blot analysis, as indicated. The results are representative of three independent experiments. (**c**) Lysates from 293T cells transfected with HA-NIK and Flag-NDRG2 were subjected to Western bolt analysis, as indicated. The results are representative of three independent experiments. (**d**) Cells were transfected with HA-NIK, Flag-NDRG2, and pNF-κB-Luc and pRL-TK plasmids and then subjected to NF-κB reporter assays. The data are expressed as the mean ± s.d. Student’s t-test was used for the statistical analysis (p < 0.05). (e) The lysates of HUT102-parental, HUT102-Mock and HUT102-NDRG2 cells were immunoprecipitated with anti-Flag and NIK antibodies and assayed by Western blotting with the indicated antibodies. The lysates of 293T cells transfected with HA-NIK and Flag-NDRG2 were immunoprecipitated with anti-Flag and HA antibodies and assayed by Western blotting with the indicated antibodies. The results are representative of three independent experiments.

Because we recently demonstrated that NDRG2 recruits PP2A to facilitate PTEN dephosphorylation, resulting in the suppression of phosphorylated AKT^15^, NDRG2 might have a function as a PP2A recruiter to inhibit the non-canonical pathway in ATL cells. Therefore, to assess whether PP2A might be involved in the suppression of the non-canonical pathway, we measured the activation of PI3K/AKT and the processing of p52 after treatment with or without dose-dependent okadaic acid (OA), an inhibitor of the serine/threonine protein phosphatases PP1 and PP2A, in two HTLV-1+ cell lines (HUT102 and KOB). PTEN-Ser380/Thr382/Thr383 and AKT-Ser473 phosphorylation and p52 processing were increased by OA in a dose-dependent manner (Supplementary S4a and b). In NDRG2-expressing HTLV-1+ cells, the levels of phosphorylated PTEN, AKT, IKKα/β and IκB and of p52 processed from p100 were significantly decreased, and the level of IκBα was increased; however, treatment with 1 μM of OA inhibited the dephosphorylation of PTEN, AKT, IKKα/β and IκBα and increased the levels of IκBα and p52 processed from p100 (Fig. 4a and Supplementary S4c). Therefore, PP2A may have an important role in the canonical and non-canonical NF-κB pathways.

In HTLV-1+ cells, NIK was overexpressed to activate the non-canonical pathway by down-regulating the negative regulator miR31^14^. Furthermore, NIK kinase activity might be regulated by the phosphorylation of threonine 559 (Thr559) in the activation loop of its kinase domain^29^,^30^. We investigated whether the total and Thr559-phosphorylated forms of NIK were increased in HTLV-1+ cells and whether the phosphorylation status of NIK is regulated by NDRG2 via PP2A. Because NIK protein turnover occurs too quickly to detect NIK protein levels in normal culture conditions, we performed western blots under the proteasome inhibitor MG132 to inhibit NIK protein degradation. The NIK protein levels in four HTLV-1+ cell lines (MT2, HUT102, KOB and KK1) were higher than those in two non-HTLV-1 leukemia cell lines (Jurkat and MOLT4) (Supplementary Fig. S4d), and the level of p52 processed from p100 in the non-canonical pathway and the phosphorylation level of NIK-Thr559 were higher in the ATL cell lines compared with those in the Jurkat and MOLT4 cell lines (Supplementary Fig. S4d and S4e). Therefore, the non-canonical NF-κB pathway is also activated through NIK overexpression with high levels phosphorylation at Thr559 in many type of cancer, as previously indicated.

In the next experiment, we assessed whether NDRG2 expression might affect the phosphorylation status of NIK along with activation of the non-canonical pathway in three HTLV-1 cell lines (HUT102, KOB and KK1). The phosphorylation of NIK-Thr559 was significantly decreased in NDRG2-expressing cells, although the NIK protein level was maintained under the introduction of NDRG2 (Fig. 4b and Supplementary Fig. S4f). To confirm the experiment, we co-transfected 293T cells with the NIK-expressing plasmid with or without the NDRG2-expressing plasmid and assessed the phosphorylation status of NIK-Thr559 and the processing of p52 from p100. While NIK expression induced the phosphorylation of NIK-Thr559 and the processing of p52 from p100 and enhanced the NF-κB transcription activity by four fold, co-transfection of NDRG2 and NIK suppressed the NIK-derived activation of each step in the non-canonical NF-κB pathway (Fig. 4c,d).

To confirm the direct binding of NIK to NDRG2, co-immunoprecipitation of endogenous NIK with transfected Flag-NDRG2 and western blotting were performed in HUT102 cells treated with MG132, using anti-Flag and anti-NIK antibodies, and vice versa. The same experiment was performed in 293T cells by co-transfecting FLAG-tagged NDRG2 and HA-tagged NIK. In each experiment, NIK and NDRG2 could be co-immunoprecipitated in both cell lines (Fig. 4e).

**Figure 5:**
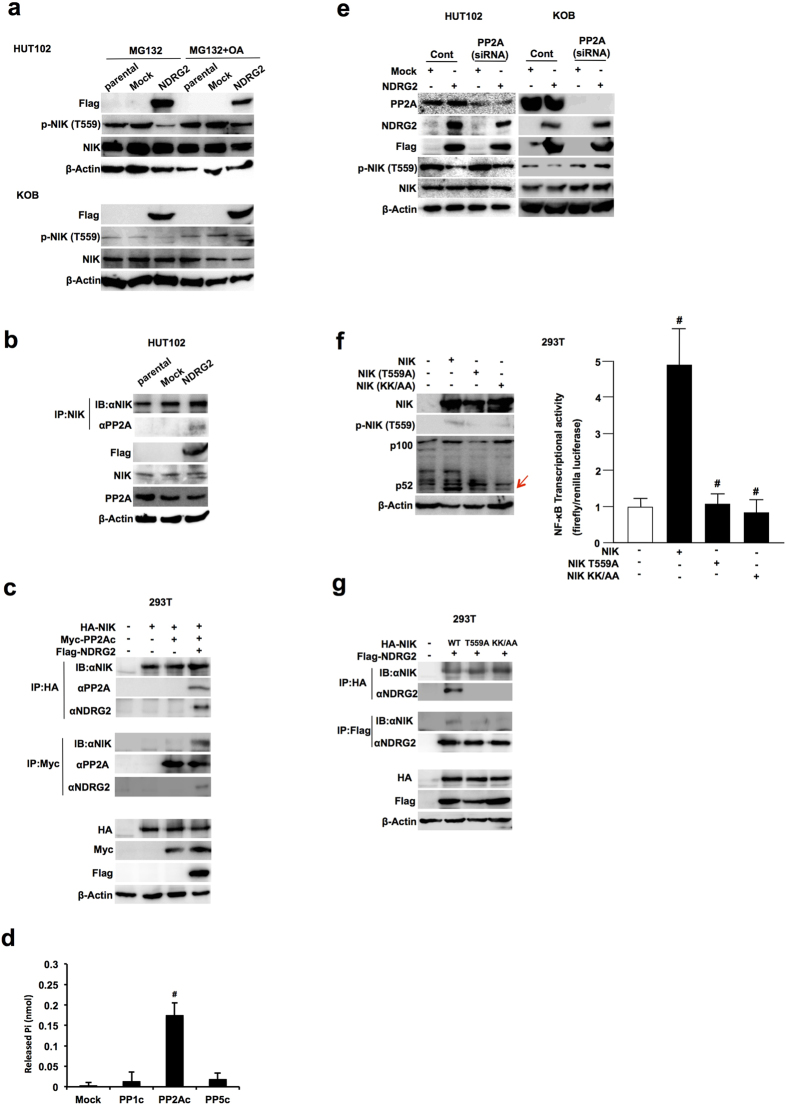
Suppression of the non-canonical NF-κB pathway through the dephosphorylation of NIK by the NDRG2/PP2A complex. (**a**) Cells were pretreated with or without MG132 (5 μM) and Okadaic acid (1 μM), and incubated with 10% FBS DMEM for another 24 h, followed by western blot analysis, as indicated. The results are representative of three independent experiments. (**b**) The lysates of HUT102-parental, HUT102-Mock and HUT102-NDRG2 cells were immunoprecipitated with an anti-NIK antibody and assayed by Western blotting with the indicated antibodies. The results are representative of three independent experiments. (**c**) The lysates of 293T cells transfected with HA-NIK, Myc-PP2A and Flag-NDRG2 were immunoprecipitated with anti-HA and Myc antibodies and assayed by Western blotting with the indicated antibodies. The results are representative of three independent experiments. (**d**) After anti-Myc immunoprecipitation from 293T cells transfected with Myc-tagged PP1c, PP2Ac, or PP5c, beads were incubated with NIK-Thr559 phosphopeptide, and phosphatase release was measured. The data are expressed as the mean ± s.d. Student’s t-test was used for the statistical analysis (p < 0.05). (**e**) HUT102-Mock, HUT102-NDRG2, KOB-Mock and KOB-NDRG2 cells were transiently transfected with control and PP2Acα siRNA, and these lysate was subjected to western blot analysis for PP2Ac, NDRG2 (Flag), total NIK and phosphorylated NIL (Thr559). (**f**) The lysates of 293T cells transfected with HA-NIK, T559A, KK/AA and Flag-NDRG2 were immunoprecipitated with anti-HA and anti-Flag antibodies and assayed by Western blotting with the indicated antibodies. The results are representative of three independent experiments. Cells were transfected with HA-NIK, T559A, KK/AA, Flag-NDRG2, and NF-κB-Luc and pRL-TK plasmids and then subjected to NF-κB reporter assays. The data are expressed as the mean ± s.d. Student’s t-test was used for the statistical analysis (p < 0.05). (**g**) The lysates of 293T cells exogenously transfected with HA-NIK, T559A, KK/AA and Flag-NDRG2 were immunoprecipitated with anti-HA and anti-Flag antibodies and assayed by Western blotting with the indicated antibodies. The results are representative of three independent experiments.

In the next experiment, to identify the serine/threonine phosphatase for NIK-Thr559, we assessed whether treatment with the phosphatase inhibitor OA modulates the dephosphorylation reaction resulting from NDRG2 expression. In this experiment, OA treatment prevented the NIK-Thr559 dephosphorylation reaction through the overexpression of NDRG2, suggesting that PP2A or other members might regulate the phosphorylation status of NIK with NDRG2 (Fig. 5a and Supplementary Fig. S4g). Because PP2A could not directly bind to PTEN but could bind to NDRG2^15^, we next examined whether NDRG2 recruits PP2A to NIK for the dephosphorylation reaction to occur. After NIK-binding proteins were immunoprecipitated in HUT102 cells using an anti-NIK antibody, the precipitated proteins were immunoblotted using an anti-PP2A antibody. However, PP2A was not detected in HUT102 cells. Next, after transfecting the FLAG-tagged NDRG2 expression vector, NIK-binding proteins were immunoprecipitated, and PP2A was clearly detected among the NIK-binding proteins (Fig. 5b). Moreover, HA-tagged NIK, Myc-tagged PP2A and/or Flag-tagged NDRG2 were co-transfected into 293T cells in different combinations, and the protein complex was detected by each specific tagged antibody (Fig. 5c). As shown in Fig. 5c, PP2A could not be detected in the protein complex from 293T cells transfected with NIK only or from NIK- and PP2Ac-transfected cells; however, PP2A could detected in the protein complex from 293T cells transfected with NIK, PP2Ac and NDRG2 expression vectors. Therefore, NIK could not directly bind to PP2A, and NDRG2 could recruit PP2A to NIK for the dephosphorylation of NIK.

In addition, to investigate the specificity of the phosphatase to dephosphorylate NIK-Thr559 by NDRG2, Myc-tagged PP1c, PP2Ac and PP5c were immunoprecipitated from NDRG2-transfected 293T cells by an anti-Myc antibody and were used for an *in vitro* NIK dephosphorylation assay using synthetic phospho-Thr559 peptide (see Materials and Methods). Myc-tagged PP2Ac significantly dephosphorylated the phospho-Thr559 peptide; however, PP1c or PP5c could not dephosphorylate the phospho-Thr559 peptide (Fig. 5d). The small interfering RNA (siRNA)-regulated knockdown of PP2Acα suppressed the NDRG2-induced the prevention of phodphorylated NIK in HUT102 and KOB-NDRG2 cells (Fig. 5e). Furthermore, we constructed a NIK mutant by replacing Thr559 with alanine (NIK-T559A) and a kinase-dead NIK mutant by substituting two adjacent lysines in the ATP binding site of the kinase domain with alanines (NIK-KK429/430AA)^31^, and then we co-transfected 293T cells with a WT or mutant plasmid of NIK and assessed the importance of Thr559 phosphorylation for the processing of p52 from p100 and the transcriptional activity of NF-κB. Whereas NIK-WT transfection induced the phosphorylation of NIK-Thr559 and the processing of p52 from p100 and enhanced the transcription of NF-κB, transfection of the NIK-T559A or NIK-KK429/430AA mutant could not induce the activation of each step of the non-canonical NF-κB pathway (Fig. 5f). Moreover, we co-transfected 293T cells with HA-tagged NIK-WT, NIK-T559A, or NIK-KK/AA with Flag-tagged NDRG2 and assessed the binding ability of NIK to NDRG2 by immunoprecipitation. NIK-WT could bind to NDRG2 (Fig. 5g), but the NIK mutants could not, suggesting that NIK-Thr559 phosphorylation is necessary for protein binding to NDRG2. Therefore, NDRG2 could bind to phosphorylated NIK-Thr559 with PP2A, resulting in the dephosphorylation of NIK-Thr559 to inactivate NIK kinase activity. In ATL cells, the loss of NDRG2 expression enhanced NIK phosphorylation, resulting in the enhanced activation of the non-canonical NF-κB pathway along with the activation of the canonical pathway by AKT phosphorylation.

## Discussion

In this study, we showed that NDRG2 suppresses the canonical NF-κB pathway through the down-regulation of the PI3K/AKT signaling pathway in the OSCC and ATL cell lines. We also demonstrated that NDRG2 decreases the activation of the non-canonical NF-κB pathway via the dephosphorylation of NIK by the recruitment of PP2A in ATL. The loss of NDRG2 expression in cancer cells, including ATL and OSCC cells, induces constitutive activation of the NF-κB pathway, thereby contributing to tumor development.

Our previous studies demonstrated that the down-regulation of NDRG2 induced aberrant activation of the PI3K/AKT signaling pathway through the inactivation of PTEN by phosphorylation at its C-terminus, thereby resulting in significantly increased cell proliferation in ATL cells and many other types of cancer cell lines *in vitro* and tumor incidence in Ndrg2-deficient mice *in vivo*. Furthermore, the enforced expression of NDRG2 in cancer cells suppressed phosphorylated AKT via the dephosphorylation of PTEN through the recruitment of PP2A^15^. Because NDRG2 is reported to directly or indirectly modulate several signal transduction pathways such as MAPK, JAK/STAT and NF-κB^16^,^17^,^18^,^19^,^20^,^21^, we suggest that the NDRG2-PP2A complex might modulate oncogenic signaling through dephosphorylation of the key associated molecules in each signaling pathway. Among them, constitutive activation of the NF-κB pathway is the most important signaling event involved in ATL tumorigenesis, and inhibition of the NF-κB pathway results in the suppression of tumor development^4^,^5^. Because NDRG2 expression is up-regulated in response to stress such as hypoxia via HIF1α^32^,^33^, oxidative stress^34^ or NF-κB activation^35^,^36^, NDRG2 might regulate the aberrant signaling pathway by several stimuli through the up-regulation of NDRG2 expression. Identification of the detailed molecular mechanism of NDRG2 will lead to remarkable progress in the understanding of tumor pathogenesis.

The canonical and non-canonical NF-κB pathways are persistently activated in cancer cells through IKK and NIK^6^,^7^,^37^,^38^,^39^, although the molecular mechanisms of IKK and NIK activation are not completely understood. Our study demonstrated that while the canonical NF-κB pathway was up-regulated in OSCC and ATL cells along with the phosphorylation of IKK and the degradation of IκBα, the non-canonical NF-κB pathway was activated in ATL cells along with the processing of p52 from p100 due to low or no p100 expression in OSCC cells. Because AKT activation plays an important role in the phosphorylation of IKK and the activation of canonical NF-κB^8^,^9^,^10^,^11^,^12^ and NDRG2 suppressed AKT phosphorylation through the dephosphorylation of PTEN, we speculate that NDRG2 regulates the canonical NF-κB pathway through the inactivation of the PI3K/AKT signaling pathway. Although the canonical NF-κB pathway was remarkably activated in OSCC and ATL cells, the overexpression of NDRG2 reduced phosphorylated IKK, followed by the suppression of IκBα degradation and NF-κB transcriptional activity. Furthermore, the PI3K inhibitor suppressed IKK phosphorylation and each step of the canonical NF-κB pathway, and the PP2A inhibitor Okadaic acid inhibited NDRG2-induced the suppression of phosphorylated AKT, PTEN and IKK, suggesting that the PI3K/AKT signaling pathway is upstream of IKK and the canonical NF-κB pathway and that NDRG2 suppresses the canonical NF-κB pathway through the inhibition of the PI3K/AKT pathway by the recruitment of PP2A.

In the non-canonical NF-κB pathway, NIK directly regulates the non-canonical NF-κB pathway through the phosphorylation of IKK and the processing of p52 from p100. The activation of NIK was regulated by phosphorylation through TAK1 and Cot^40^,^41^, although it is not clear which phospho sites are targeted. Furthermore, NIK at Thr559 was phosphorylated by NIK-NIK oligomerization, resulting in the induction of the NF-κB pathway^29^. We identified high expression of NIK, which was highly phosphorylated at Thr559, in ATL cell lines following treatment with the proteasome inhibitor MG132, suggesting that the non-canonical NF-κB pathway in ATL cells is regulated through the up-regulated expression of highly phosphorylated NIK. The overexpression of NIK mRNA in ATL cells was due to the down-regulation of miR-31^14^. Because the NIK-T559A and the kinase-dead NIK-KK/AA mutants failed to activate IKK and the NF-κB pathway, NIK phosphorylation plays an important role in regulating the non-canonical NF-κB activation^29^. Whereas NIK expression was not changed by the overexpression of NDRG2 in ATL cell lines, its phosphorylation of Thr559 was suppressed in NDRG2-expressing ATL cells. Moreover, PP2A binds to NIK in the presence of NDRG2, resulting in the direct dephosphorylation of NIK-Thr559 followed by the suppression of the processing of p52 from p100 and the transcriptional activity of NF-κB. Because PP2A is a serine/threonine phosphatase that regulates several substrates and subsequently modulates oncogenic signaling pathways, the PP2A-NDRG2 complex might regulate a novel upstream kinase of PTEN and NIK, thereby contributing to the suppression of tumor development through inhibition of the NF-κB pathway (Supplementary Fig. 5S).

Because NDRG2 is reported to suppress several signaling pathway such as PI3K/AKT, MAPK, JAK/STAT and NF-κB, we suggest that the NDRG2-PP2A complex might be involved in the dephosphorylation of various important kinases regulating each of the signaling pathways. Among them, the main function of NDRG2/PP2A is suggested to be regulating the PTEN phosphorylation status because the phenotype of NDRG2-deficient mice was similar to that of PTEN-deficient mice and AKT signaling may affect NF-κB signaling or other signaling pathways. Moreover because NDRG2 is thought to be a stress responsive gene, similarly to genotoxic p53, hypoxic HIF1α and others, NDRG2 might suppress excessively activated signaling pathways after responding to stress signaling to recover the body to its normal state. In conclusion, this study indicates that NDRG2 suppresses the canonical and non-canonical NF-κB pathways through the dephosphorylation of PTEN and NIK by recruiting PP2A. The identification of the functions of NDRG2 provides a powerful tool to understand the molecular mechanism of cancer development under stress conditions and to develop clinical applications for cancer diagnosis and therapy.

## Materials and Methods

### Reagents

LY294002, Wortmannin and Lipopolysaccharide (LPS) were obtained form Sigma-Aldrich (St. Louis, MO), MG132 was obtained from LifeSensors (Malvern, PA) and Okadaic Acid (OA) and Tumor Necrosis Factor α (TNFα) were obtained from Wako (Osaka, Japan). The transfection reagent HilyMax and the cell proliferation/cell toxicity kit Cell Counting Kit-8 were purchased from DOJINDO (Kumamoto, Japan). Primary antibodies were purchased as follows: goat polyclonal antibodies against NDRG2 (E20) and phospho-NIK (Thr559), rabbit polyclonal antibodies against IκBα (C-21), NEMO (FL-419), and NIK (H-248), and a mouse monoclonal antibody against p100/p52 (C-5) from Santa Cruz (Santa Cruz, CA); rabbit polyclonal antibodies against AKT (9272), phospho-AKT (Ser473) (D9E), PTEN (138G6), and phospho-PTEN (Ser380/Thr382/Thr383) (9554), rabbit monoclonal antibodies against phospho-IKKα/β (Ser176/180) (16A6), GSK3β (27C10), phospho-GSK3β (Ser9) (D85E12) and PP2A C Subunit (PP2Ac) (52F8), and a mouse monoclonal antibody against phospho-IκBα (Ser32/36) (5A5) from Cell Signaling Technology (Danvers, MA); and mouse monoclonal antibodies against β-actin (AC-15) and Flag M2 (F3165) from Sigma-Aldrich. The mouse monoclonal antibody against Tax (MI73) was a kind gift from Dr. M. Matsuoka (Kyoto University, Japan).

### Plasmids and siRNA treatment

The Flag-tagged NDRG2 expression vector (NDRG2/pCMV26) has been described elsewhere^15^. Hemagglutinin (HA)-tagged NIK (HA-NIK), NIK-KK429/430AA, the NF-κB firefly luciferase reporter (NF-κB-Luc) and the Renilla luciferase expression plasmid (TK-Luc) were kind gifts from Dr. H. Iha (Ohita University, Japan). To generate the substitution mutant NIK expression vector (NIK-T559A), PCR-based mutagenesis was performed to induce the mutation in the NIK coding sequence using mutagenic primers. The small interfering RNA (siRNA) of PP2Acα (sc-43509) and control (6568) were purchased from Santa Cruz and Cell Signaling Technology, respectively^15^. The transfections were performed using Amaxa cell line Nucleofector kit V (LONZA, Germany) and HilyMax, following the manufacturer’s protocol.

### Patient samples

Blood samples were obtained with informed consent with approval by the Institutional Review Board of the Faculty of Medicine, University of Miyazaki. The collection of ATL cells from the patients and CD4+ lymphocytes from volunteers was performed as described previously^15^.

### Tissue collection

The Ndrg2-deficient mice were previously described^15^. Mouse peripheral blood mononuclear cell (PBMC) was isolated from wild-type mice by direct cardiac puncture. The lymphoma from Ndrg2-deficient mice was homogenized in Laemmli SDS sample buffer (62.5 mM Tris-HCl, pH 6.8, 2% SDS, 25% glycerol, 5% β-mercaptoethanol, and 0.01% bromophenol blue), and the protein extracts were subjected to western blot. The animal experiments were performaed in accordance with the protocols approved by the Animal Experiment Review Board of the University of Miyazaki.

### Cell culture

Jurkat and MOLT4 are HTLV-1-negative human T-ALL cell lines. KOB and KK1 are IL2–dependent ATL cell lines. SU9T-01, ED and S1T are IL2-independent ATL cell lines. MT2 and HUT102 are human T-cell lines transformed by HTLV-1 infection. Jurkat and MOLT4 cells were obtained from the Fujisaki Cell Center, Hayashibara Biochemical Laboratories (Okayama, Japan). MT2 and HUT102 cells were a kind gift from Dr. Kuan-Teh. Jeang (National Institutes of Health, USA). KOB and KK1 cells were kind gifts form Dr. Y. Yamada (Nagasaki University, Japan). SU9T-01 and S1T cells were kind gifts from Dr. N. Arima (Kagoshima University, Japan). ED was a kind gift from Dr. M. Maeda (Kyoto University, Japan). Human embryonic kidney 293T cells were obtained from RIKEN Bioresource Center (Tsukuba, Japan). IL2-dependent ATL cell lines were maintained in PRMI1640 medium supplemented with 10% fetal bovine serum (FBS) and 50 JRU per ml recombinant human IL2 (Takeda) in a humidified atmosphere of 5% CO_2_ at 37 °C. The HTLV-1 negative cell lines, IL2-independent ATL cell lines, and cell lines transfected with HTLV-1 were maintained in the same medium without IL2. Other cells were maintained in Dulbecco’s modified Eagle’s medium (DMEM, Wako, Japan) supplemented with 10% FBS.

### Western blot

Cells were harvested from 6-well plates. For the extraction of protein in tissues, excised organs were homogenized in NP-40 lysis buffer (50 mM Tris-HCl, pH8.0, 150 mM NaCl, 5 mM EDTA, 1% NP-40) supplemented with a proteinase inhibitor cocktail (Sigma-Aldrich) and phosphatase inhibitor tablet (PhosStop, Roche). The lysate was centrifuged for 10 min at 15000 x g (maximum) at 4 °C, and the supernatant was then collected. The protein concentration was determined by BCA protein assay (Thermo SCIENTIFIC, Waltham, MA) with bovine serum albumin (BSA) standard. Equal amounts of protein samples were loaded, separated by SDS-polyacrylamide gel electrophoresis and then transferred to a polyvinylidene difluoride membrane (PVDF, Immobilon-P, Millipore). The membranes were blocked in PBS–Tween (0.1%) with 1% BSA or 5% nonfat dried milk and were then probed with the primary antibodies diluted PBST-BSA, 5% nonfat dried milk or Can Get Signal Buffer (TOYOBO, Japan). The bands were detected using Lumi-light Plus kit (Roche) and LAS-3000. Band intensities were quantified with the NIH Image J software. All primary antibodies were used at a dilution of 1:1000.

### Immunoprecipitation

The lysates were incubated with 1 μg of the indicated antibodies or normal IgG with constant rotation at 4 °C overnight and were then incubated with Protein G Sepharose 4 Fast Flow (GE Healthcare, Uppsala, Sweden) or Flag M2 Affinity Gel (Sigma-Aldrich) for 2 h. The immunoprecipitates were washed 3 times with PBS, and the bound proteins were denatured with SDS sample buffer. Each sample was subjected to western blotting.

### Reporter luciferase assay

Cells were transfected with pNF-κB-Luc and pRL-TK vectors using Amaxa or HillyMax. The NF-κB transcriptional activity was measured using a dual luciferase assay kit (Promega, Madison, WI) according to the manufacturer’s instructions with a luminometer (Turner Designs TD-20/20, Promega).

### cDNA synthesis, reverse transcription (RT)-PCR and quantitative real-time PCR

Total RNA was isolated from cells using TRIzol reagent (Invitrogen). The cDNA was synthesized from 1 μg RNA with the RNA PCR kit with oligo(dT) primers (TaKaRa) and was then used as a template for RT-PCR analysis. Quantitative real-time PCR (qPCR) was carried out using the Applied Biosystems StepOne Real Time PCR System (Applied Biosystems) and the GeneAce SYBR qPCR Mixα (Nippon Gene, Japan). The expression levels of target genes were normalized by β-actin mRNA.

### Dephosphorylation assay for synthetic peptide

The NIK-Thr559 phosphopeptide (TGDYIPGpTETHMAPE) was obtained from the TORAY Research Center (Tokyo, Japan). The release of inorganic phosphates from the phosphopeptide was assessed using a malachite green assay kit (BIOMOL Green reagent, BIOMOL). Immunoprecipitated samples were incubated with 100 μM NIK-Thr559 phosphopeptide in 10 μl of phosphatase assay buffer (20 mM HEPES pH 7.0, 1 mM MnCl_2_, 8 mM MgCl_2_, 1 mM dithiothreitol and 100 μg/ml BSA) at 30 °C for 60 min. The reactions were terminated by the addition of 40 μl of TE buffer (10 mM Tris–HCl, pH 8.0, 1 mM EDTA) and 100 μl of BIOMOL Green reagent, and the absorbance was read at 620 nm after incubation for 20 min at room temperature.

### Statistical analysis

Data were presented as the means ± s.d. We used two-tailed Student’s t-tests and Mann-Whitney U-tests for comparisons within each parameter. Probability values of <0.05 were considered statistically significant.

## Additional Information

**How to cite this article**: Ichikawa, T. *et al.* Loss of NDRG2 enhanced activation of the NF-κB pathway by PTEN and NIK phosphorylation for ATL and other cancer development. *Sci. Rep.* **5**, 12841; doi: 10.1038/srep12841 (2015).

## Supplementary Material

Supplementary Information
