# Supplementary material for: Loss of NDRG2 enhanced activation of the NF-κB pathway by PTEN and NIK phosphorylation for ATL and other cancer development
Source: Sci Rep. 2015 Aug 13;5:12841. doi: 10.1038/srep12841 (PMC4534796; doi:10.1038/srep12841)
Supplement: Supplementary Information [file srep12841-s1.pdf]

Supplementary information

Loss of NDRG2 enhanced activation of the NF-κB pathway by PTEN and NIK phosphorylation for ATL and other cancer development

Tomonaga Ichikawa, Shingo Nakahata, Masahiro Fujii, Hidekatsu Iha & Kazuhiro Morishita

[1]Division of Tumor and Cellular Biochemistry, Department of Medical Sciences, University of Miyazaki, 5200 Kihara, Kiyotake, Miyazaki 889-1692, Japan.
[2]Division of Virology, Graduate School of Medical and Dental Sciences, Niigata University, Niigata 951-8510, Japan
[3]Department of Microbiology, Faculty of Medicine, Oita University, Yufu, Oita, Japan

***Corresponding author:**
Kazuhiro Morishita, MD, PhD
Division of Tumor and Cellular Biochemistry, Department of Medical Sciences, University of Miyazaki, 5200 Kihara, Kiyotake, Miyazaki 889-1692, Japan.
Email: kmorishi@med.miyazaki-u.ac.jp

Supplementary Figures S1-S5

Supplementary Figure 1S. The activation of canonical NF-κB pathway in OSCC

(a) Stably transfected HSC3 cell lines were cultured with serum-free DMEM for 24 h, and the quiescent cells were treated with LPS (1 μg/ml) and TNFα (5 ng/ml) in serum-free medium as indicated, and subjected to Western bolt analysis as indicated. Results are representative of three independent experiments. (b) The graph shows relative band intensity of p-IKK, p-IκBα and IκBα in SAS. The relative density of the bands is normalized to β-Actin. (c) The graph shows relative band intensity of p-IKK, p-IκBα and IκBα in HSC3. The relative density of the bands is normalized to β-Actin. (d) OSCC cell lines were subjected to western blot analysis of p100/p52. (e) Cytosolic and nuclear proteins were subjected to Western bolt analysis as indicated. The results are representative of three independent experiments. (f) Cells were transfected with pNF-κB-Luc and pRL-TK plasmid, stimulated with LPS and TNFα, and then subjected to NF-κB reporter assays. The data are expressed as the mean ± standard deviation (s.d). Student's t-test was used for the statistical analysis ($p<0.05$). (g) Stably transfected SAS cell lines were cultured with serum-free DMEM for 24 h, and the quiescent cells were treated with LPS (1 μg/ml) and TNFα (5 ng/ml) in serum-free medium as indicated, and subjected to Western bolt analysis of PI3K/AKT signaling pathway. Results are representative of three independent experiments. (h) Reverse-transcription PCR (RT-PCR) analysis of inflammatory-related genes in LPS (1 μg/ml) and TNFα (5 μg/ml)-treated SAS. (i) Cells were transfected with pNF-κB-Luc and pRL-TK plasmids, pretreated with or without LY294002 (5 μM) in serum-free DMEM, stimulated with LPS and TNFα, and then subjected to NF-κB reporter assays. The data are expressed as the mean ± s.d. Student's t-test was used for the statistical analysis ($p<0.05$)

a

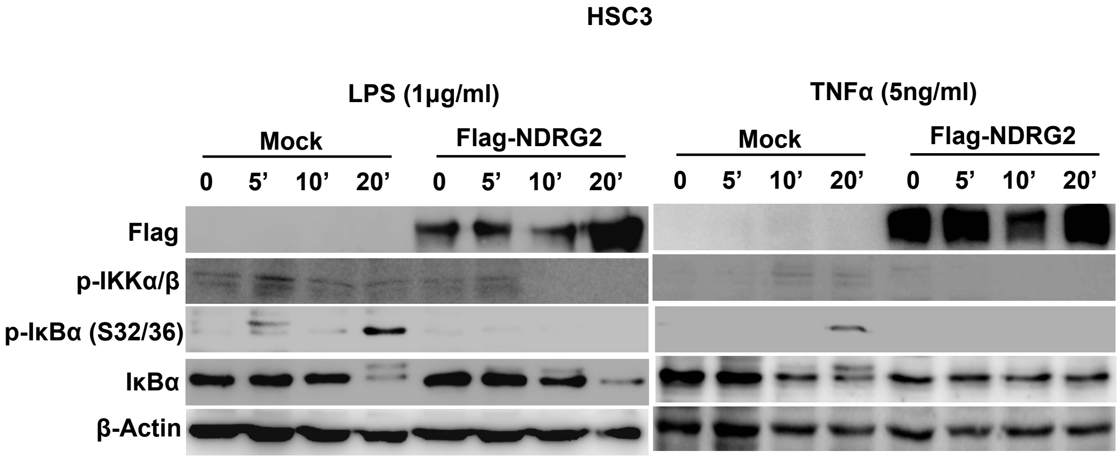
HSC3
LPS (1μg/ml)
TNFα (5ng/ml)
Mock
Flag-NDRG2
Mock
Flag-NDRG2
0
5’
10’
20’
0
5’
10’
20’
0
5’
10’
20’
0
5’
10’
20’
Flag
p-IKKα/β
p-IκBα (S32/36)
IκBα
β-Actin

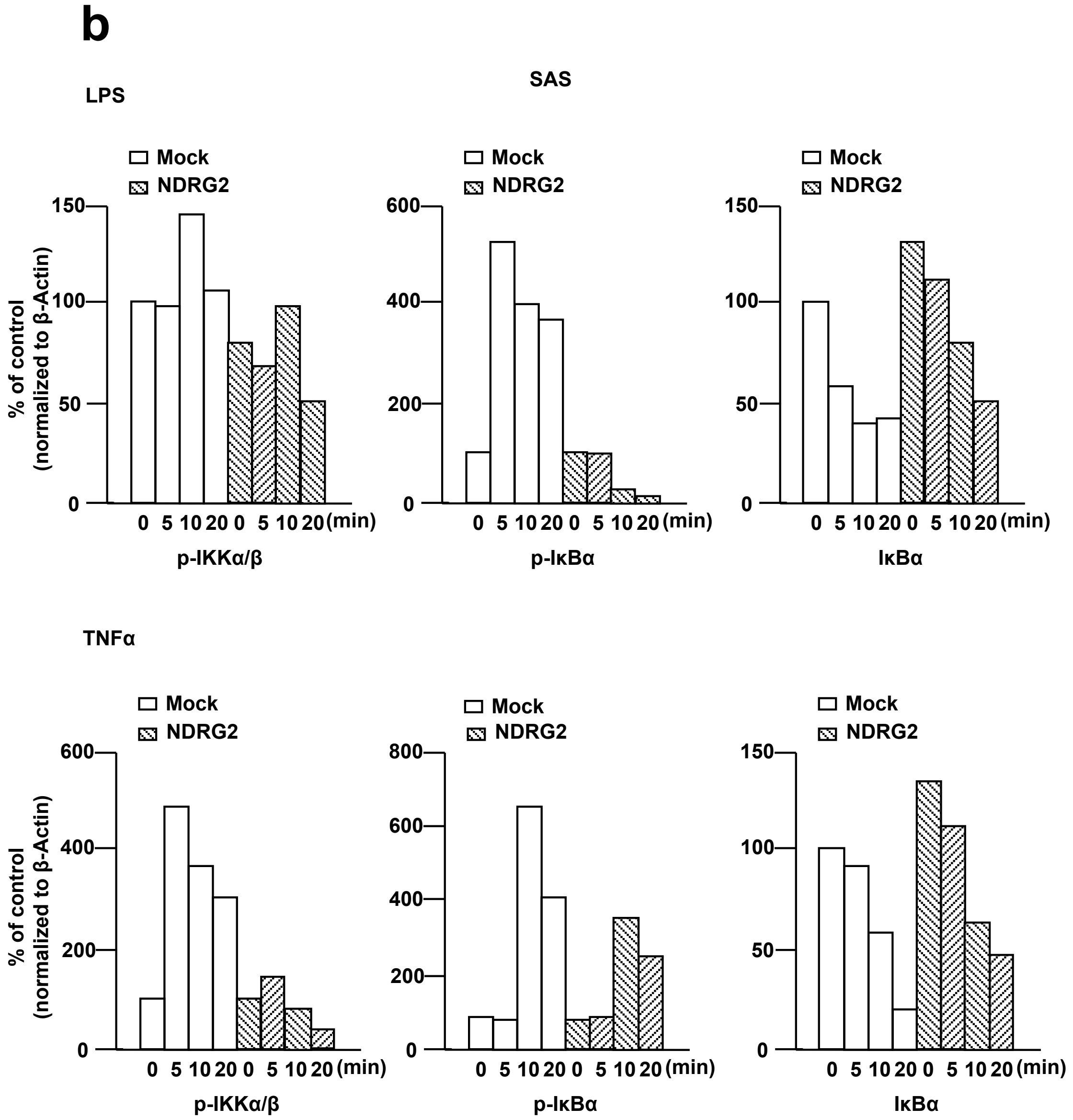
b
SAS
LPS
Mock
NDRG2
% of control
(normalized to β-Actin)
150
100
50
0
600
400
200
0 5 10 20 0 5 10 20 (min)
p-IKKα/β
p-IκBα
IκBα
TNFα
800

c

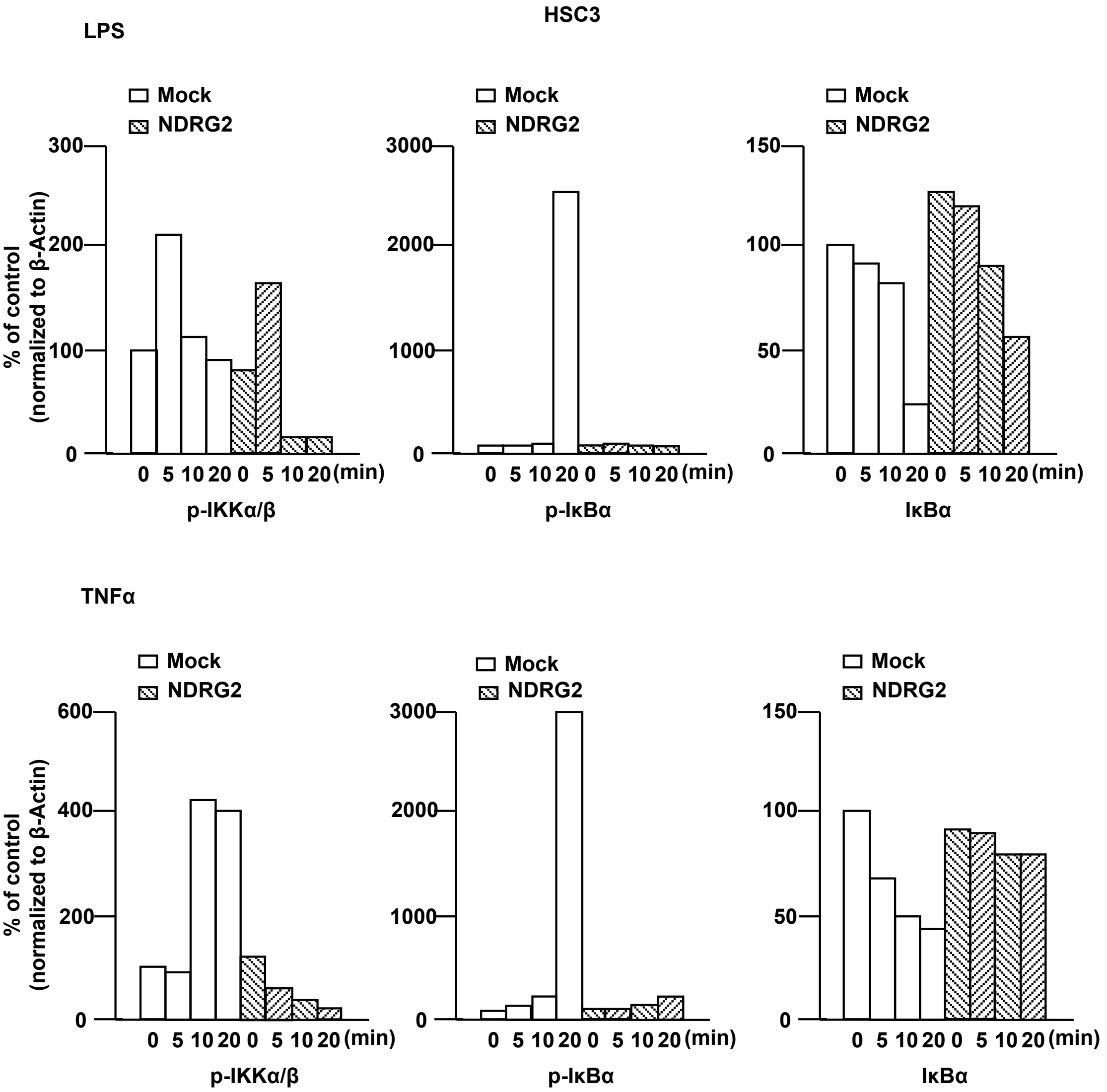

HSC3
LPS
TNFα
Mock
NDRG2
% of control (normalized to β-Actin)
0 5 10 20 0 5 10 20 (min)
p-IKKα/β
p-IκBα
IκBα

d

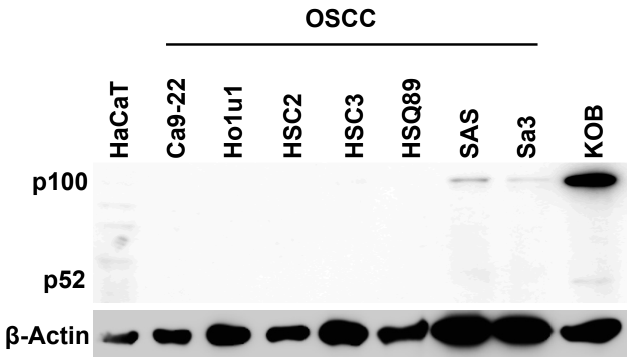

OSCC
HaCaT
Ca9-22
Ho1u1
HSC2
HSC3
HSQ89
SAS
Sa3
KOB
p100
p52
β-Actin

e

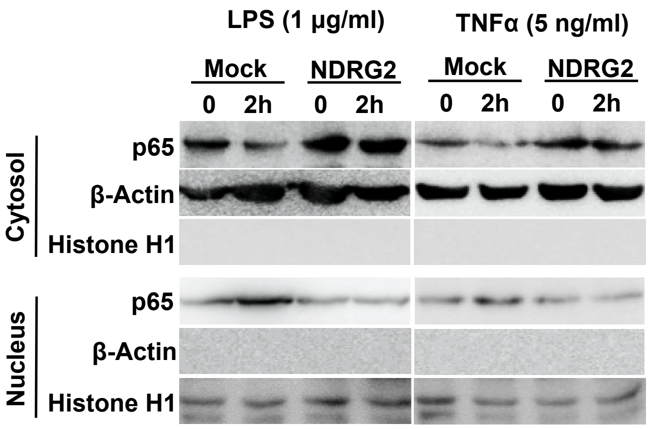

LPS (1 μg/ml)
TNFα (5 ng/ml)
Mock
NDRG2
Mock
NDRG2
0
2h
0
2h
0
2h
0
2h
Cytosol
p65
β-Actin
Histone H1
Nucleus
p65
β-Actin
Histone H1

f

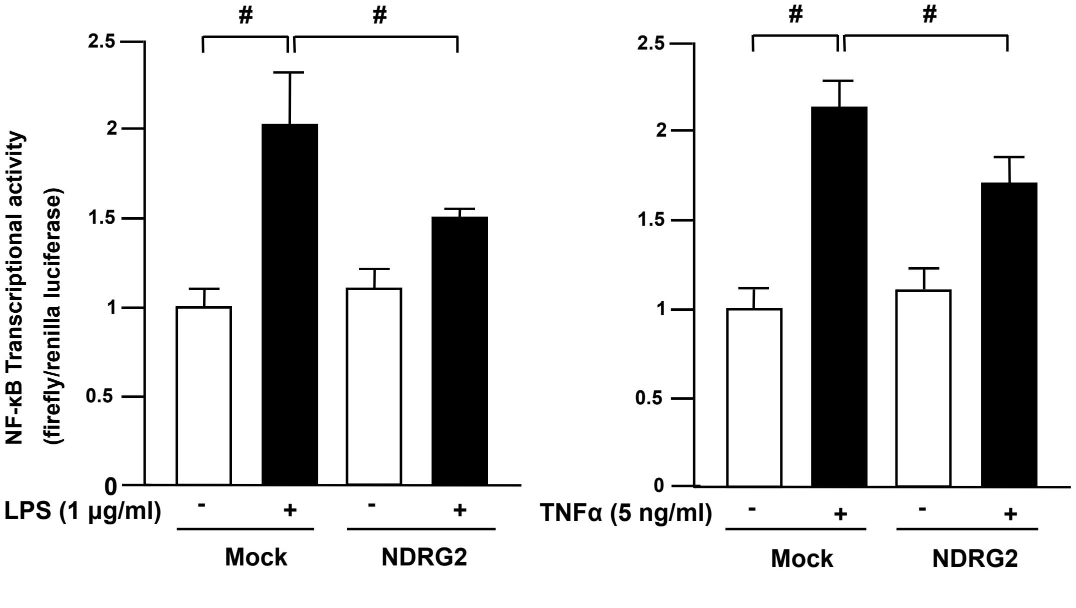

NF-κB Transcriptional activity
(firefly/renilla luciferase)
#
#
2.5
2
1.5
1
0.5
0
LPS (1 μg/ml)
-
+
-
+
Mock
NDRG2
#
#
2.5
2
1.5
1
0.5
0
TNFα (5 ng/ml)
-
+
-
+
Mock
NDRG2

g

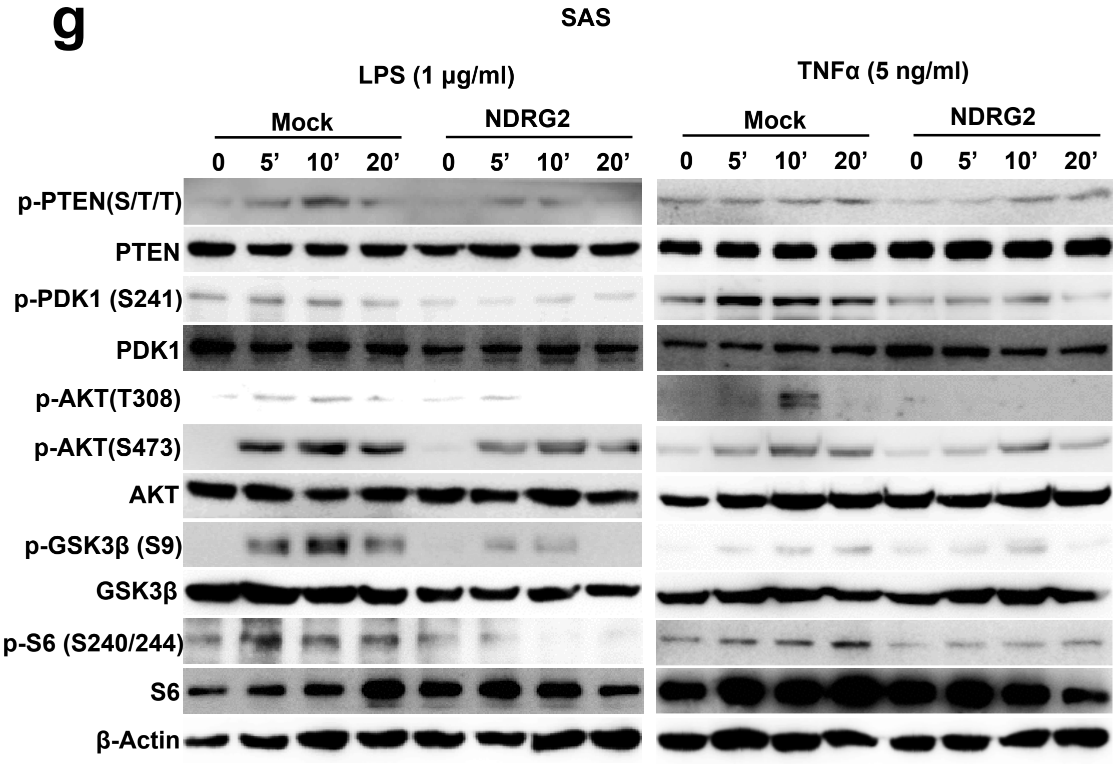
SAS
LPS (1 µg/ml)
TNFα (5 ng/ml)
Mock
NDRG2
Mock
NDRG2
0 5' 10' 20' 0 5' 10' 20' 0 5' 10' 20' 0 5' 10' 20'
p-PTEN(S/T/T)
PTEN
p-PDK1 (S241)
PDK1
p-AKT(T308)
p-AKT(S473)
AKT
p-GSK3β (S9)
GSK3β
p-S6 (S240/244)
S6
β-Actin

h

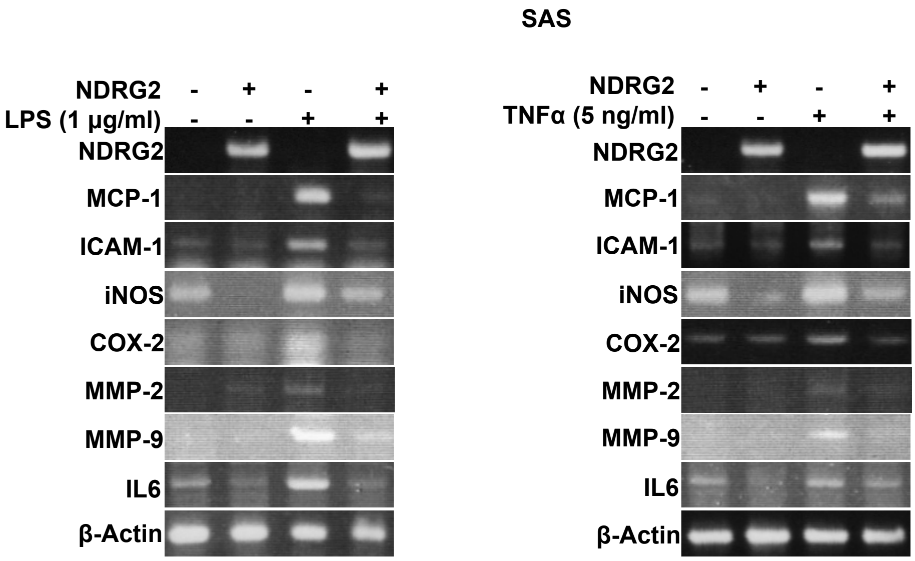
SAS
NDRG2 - + - +
LPS (1 µg/ml) - - + +
NDRG2 - + - +
TNFα (5 ng/ml) - - + +
NDRG2
MCP-1
ICAM-1
iNOS
COX-2
MMP-2
MMP-9
IL6
β-Actin

i

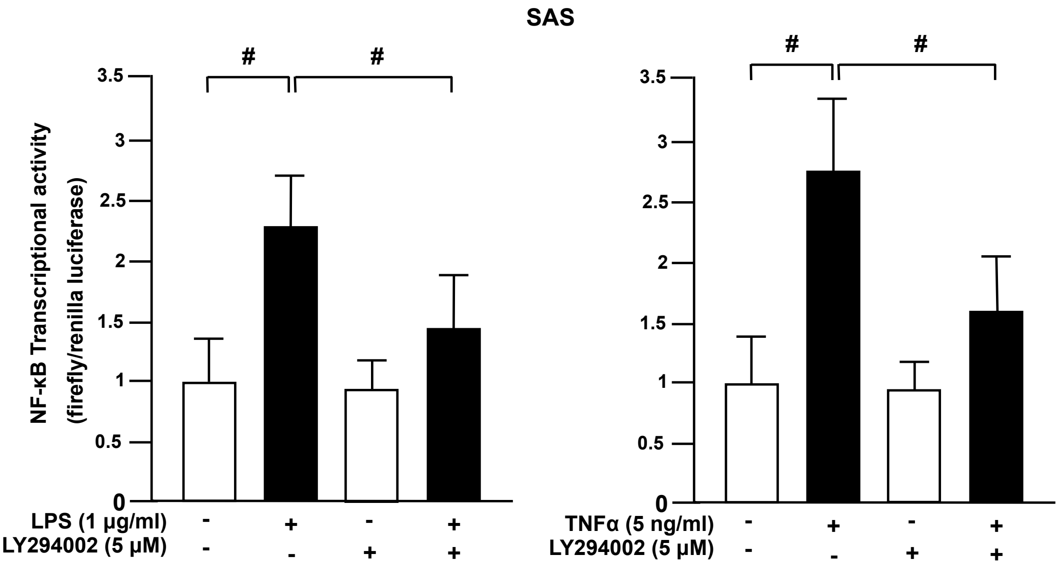
SAS
NF-κB Transcriptional activity
(firefly/renilla luciferase)
#
#
3.5
3
2.5
2
1.5
1
0.5
0
LPS (1 μg/ml) - + - +
LY294002 (5 μM) - - + +
TNFα (5 ng/ml) - + - +
LY294002 (5 μM) - - + +

Supplementary Figure 2S. The activation of canonical NF-κB pathway in MEF

(a) The graph shows relative band intensity of p-IKK, p-IκBα and IκBα in MEF. The relative density of the bands is normalized to β-Actin. (b) MEFs were transfected with pNF-κB-Luc and pRL-TK plasmids, stimulated with LPS and TNFα, and then subjected to NF-κB reporter assays. The data are expressed as the mean ± s.d. Student's t-test was used for the statistical analysis ($p<0.05$). (c) MEF cells were subjected to western blot analysis of p100/p52. (d) MEFs were transfected with pNF-κB-Luc and pRL-TK plasmids pretreated with or without LY294002 (5 μM) in serum-free DMEM, stimulated with LPS and TNFα, and then subjected to NF-κB reporter assays. The data are expressed as the mean ± s.d. Student's t-test was used for the statistical analysis ($p<0.05$).

a

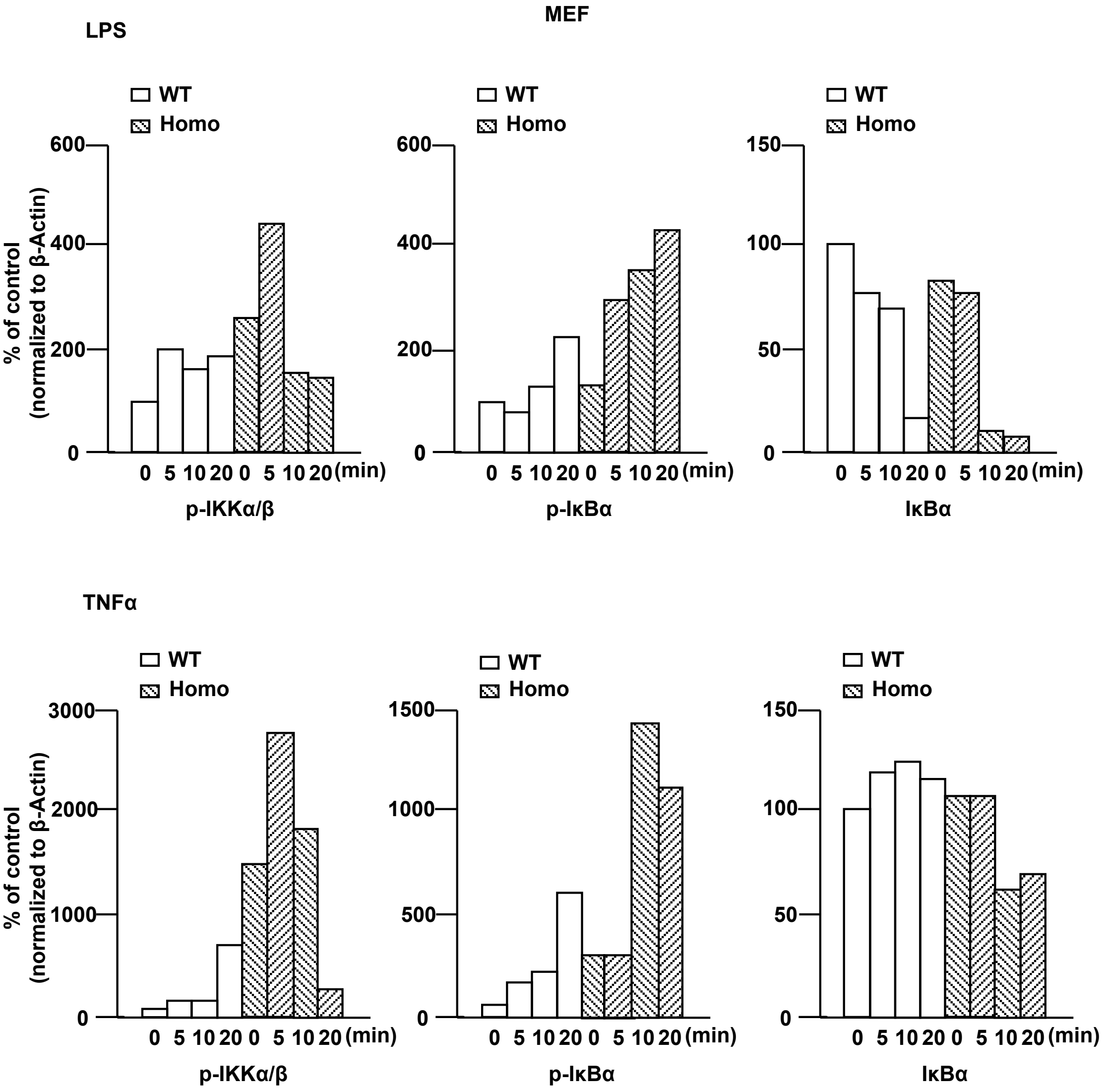

MEF
LPS
WT
Homo
% of control (normalized to β-Actin)
600
400
200
0
0 5 10 20 0 5 10 20 (min)
p-IKKα/β
p-IκBα
150
100
50
IκBα
TNFα
3000
2000
1000
1500
1000
500

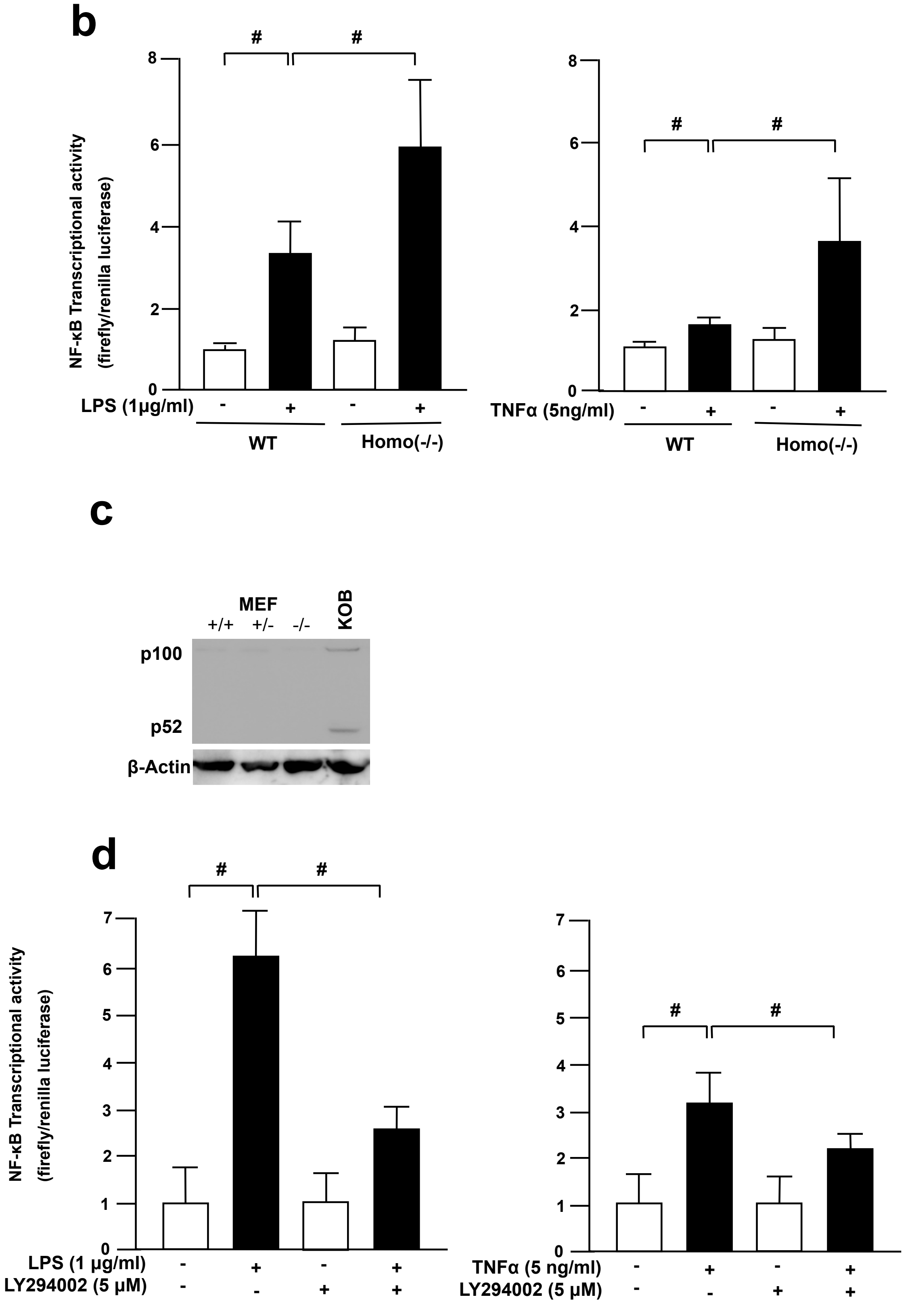
b
#
#
8
6
4
2
0
NF-κB Transcriptional activity
(firefly/renilla luciferase)
LPS (1μg/ml)
-
+
-
+
WT
Homo(-/-)
TNFα (5ng/ml)
WT
Homo(-/-)
c
MEF
+/+
+/-
-/-
KOB
p100
p52
β-Actin
d
7
5
3
1
LPS (1 μg/ml)
LY294002 (5 μM)
TNFα (5 ng/ml)

Supplementary Figure 3S. The inhibition of the NF-κB signaling pathway by NDRG2 and PI3K inhibitor in ATL

(a) The graphs show the quantification of relative band intensity of the canonical NF-κB signaling pathway in ATL cell lines. The relative density of the bands is normalized to β-Actin. The data are expressed as the mean ± s.d. Student's t-test was used for statistical analysis ($p<0.05$). n=3. (b) The graphs show the quantification of relative band intensity of the non-canonical NF-κB signaling pathway in ATL cell lines. The relative density of the bands is normalized to β-Actin. The data are expressed as the mean ± s.d. Student's t-test was used for statistical analysis ($p<0.05$). n=3. (c) The graphs show the quantification of relative band intensity of the PI3K inhibitor LY294002-treated canonical NF-κB signaling pathway in ATL cell lines. The relative density of the bands is normalized to β-Actin. The data are expressed as the mean ± s.d. Student's t-test was used for statistical analysis ($p<0.05$). n=3. (d) The graphs show the quantification of relative band intensity of the PI3K inhibitor Wortmannin-treated canonical NF-κB signaling pathway in ATL cell lines. The relative density of the bands is normalized to β-Actin. The data are expressed as the mean ± s.d. Student's t-test was used for statistical analysis ($p<0.05$). n=3. (e) The graphs show the quantification of relative band intensity of the PI3K inhibitor LY294002 or Wortmannin-treated non-canonical NF-κB signaling pathway in ATL cell lines. The relative density of the bands is normalized to β-Actin. The data are expressed as the mean ± s.d. Student's t-test was used for statistical analysis ($p<0.05$). n=3.

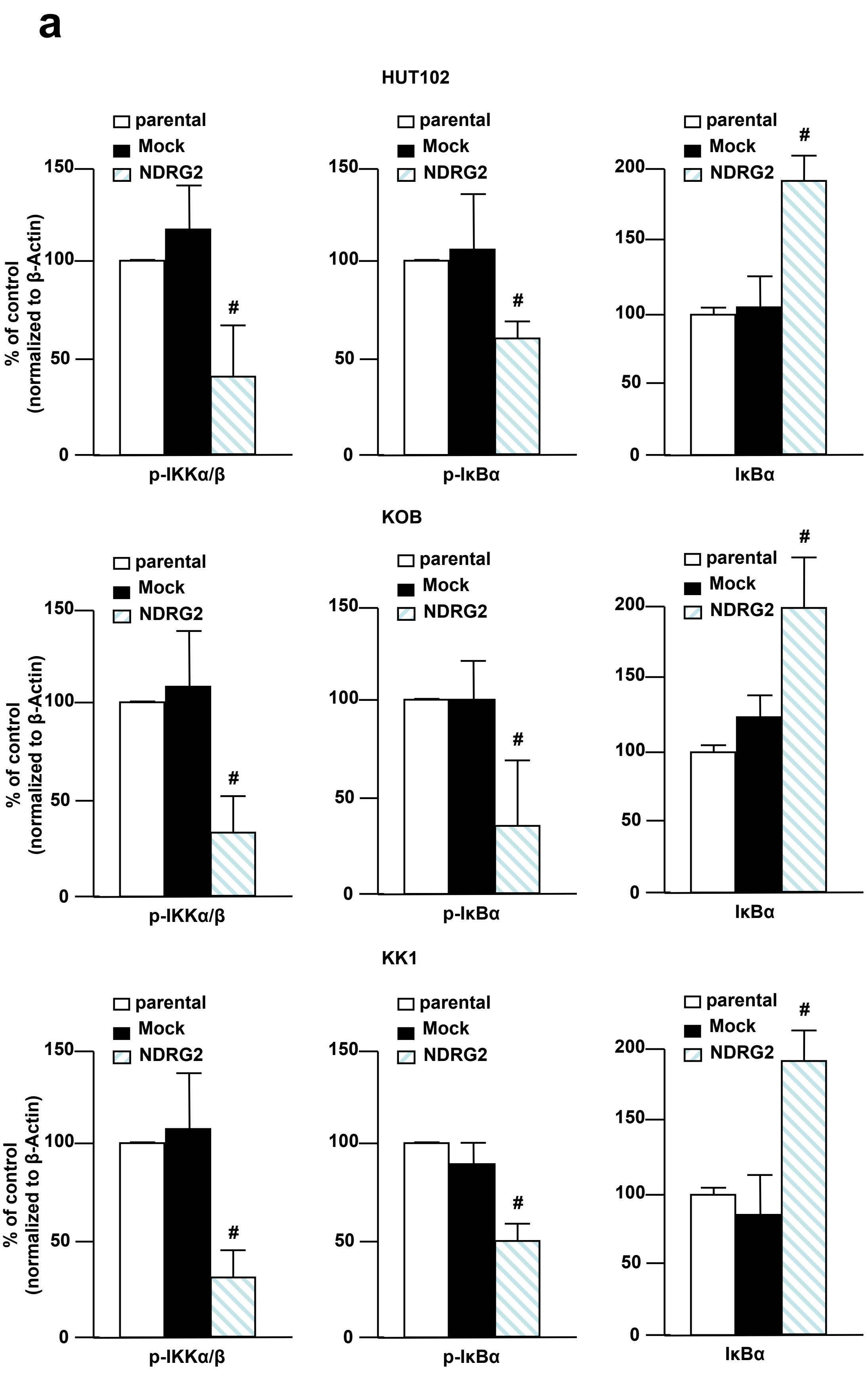

a
HUT102
KOB
KK1
parental
Mock
NDRG2
% of control
(normalized to β-Actin)
p-IKKα/β
p-IκBα
IκBα
#
0
50
100
150
200

b

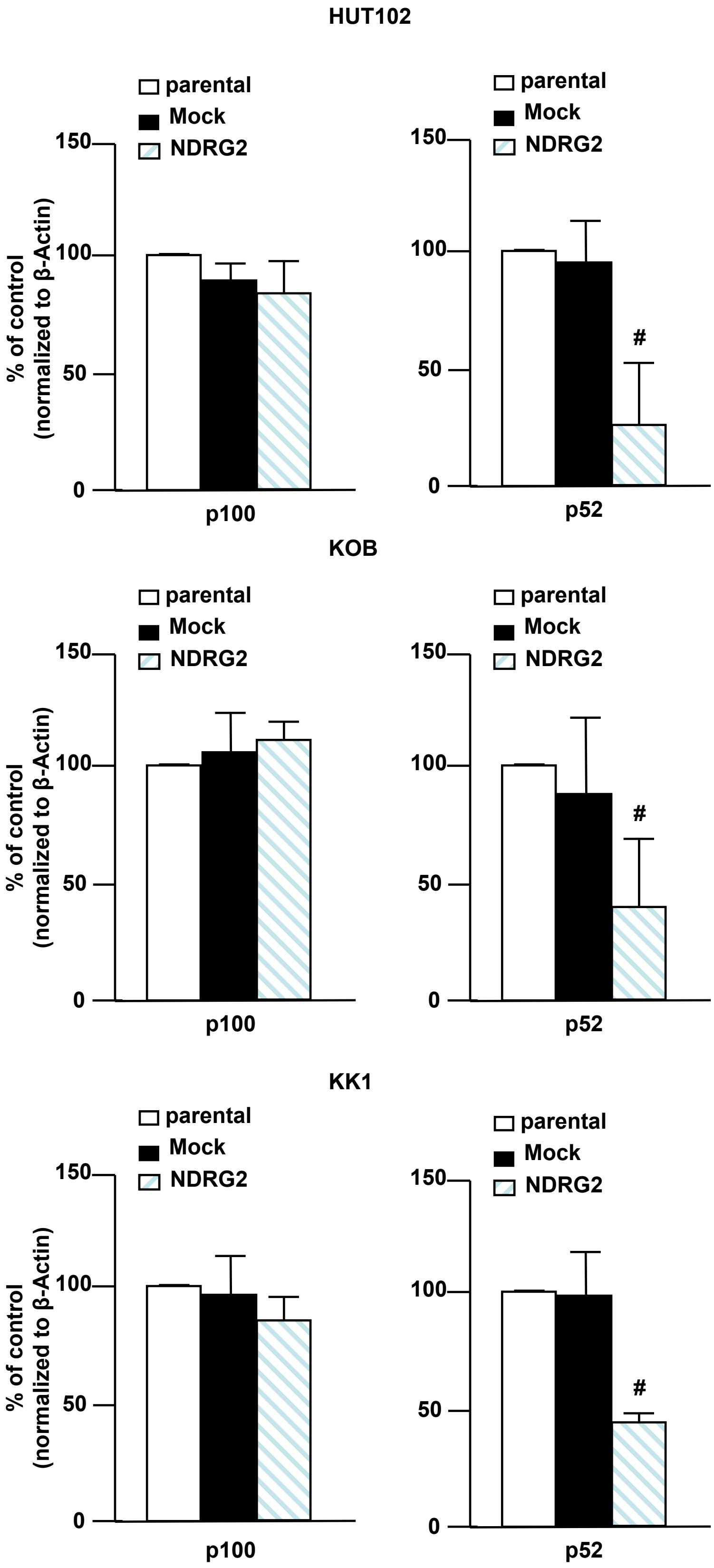
HUT102
parental
Mock
NDRG2
% of control (normalized to β-Actin)
150
100
50
0
p100
p52
#
KOB
parental
Mock
NDRG2
% of control (normalized to β-Actin)
150
100
50
0
p100
p52
#
KK1
parental
Mock
NDRG2
% of control (normalized to β-Actin)
150
100
50
0
p100
p52
#

c

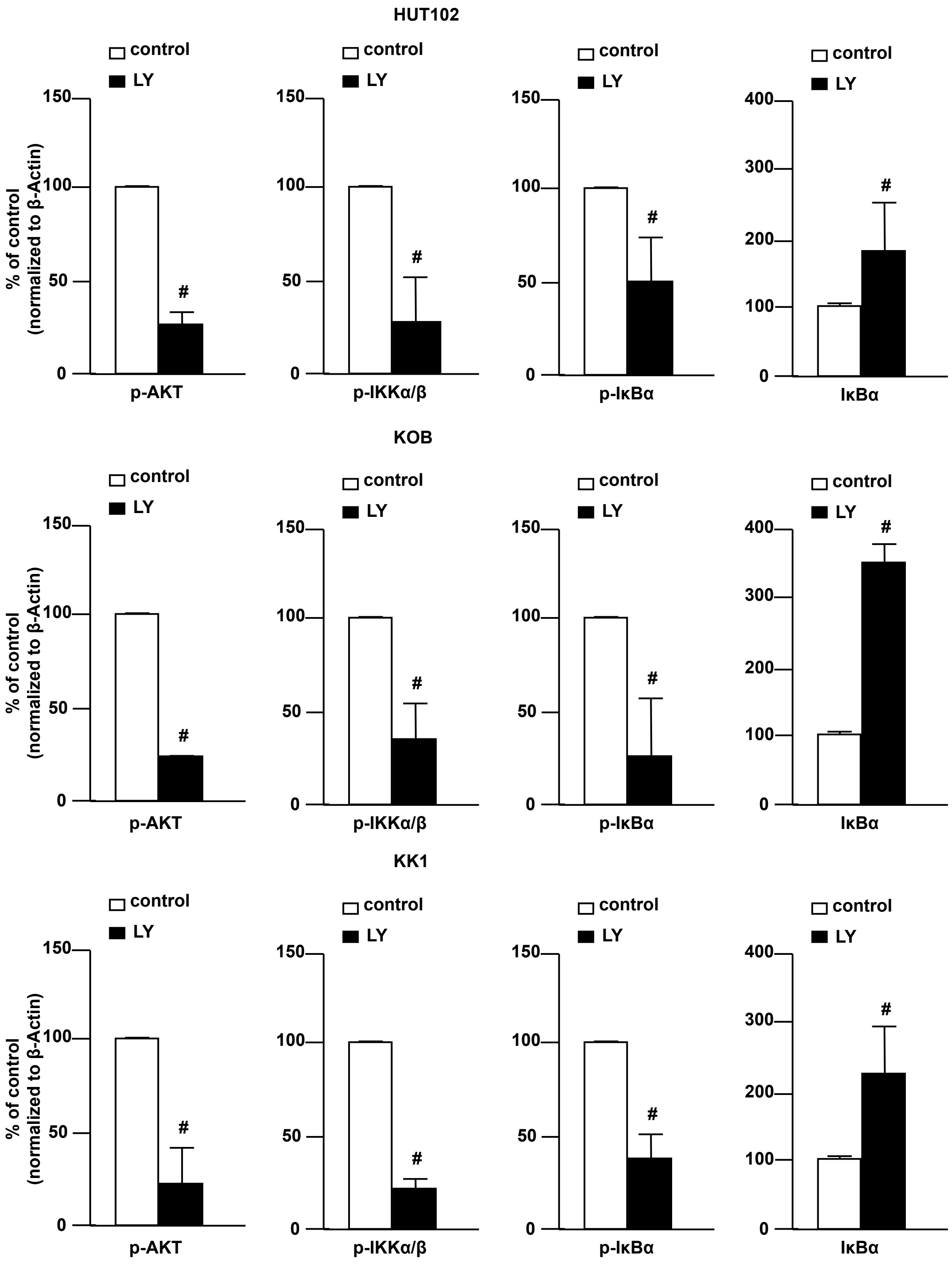
HUT102
KOB
KK1
control
LY
% of control
(normalized to β-Actin)
0
50
100
150
200
300
400
#
p-AKT
p-IKKα/β
p-IκBα
IκBα

d

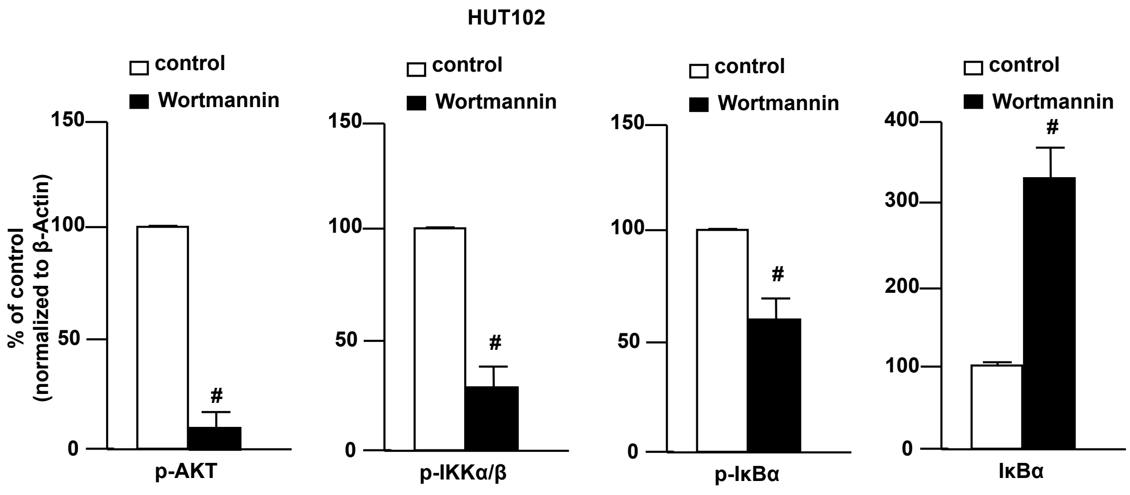

HUT102
control
Wortmannin
% of control
(normalized to β-Actin)
150
100
50
0
#
p-AKT
p-IKKα/β
p-IκBα
400
300
200
IκBα

e

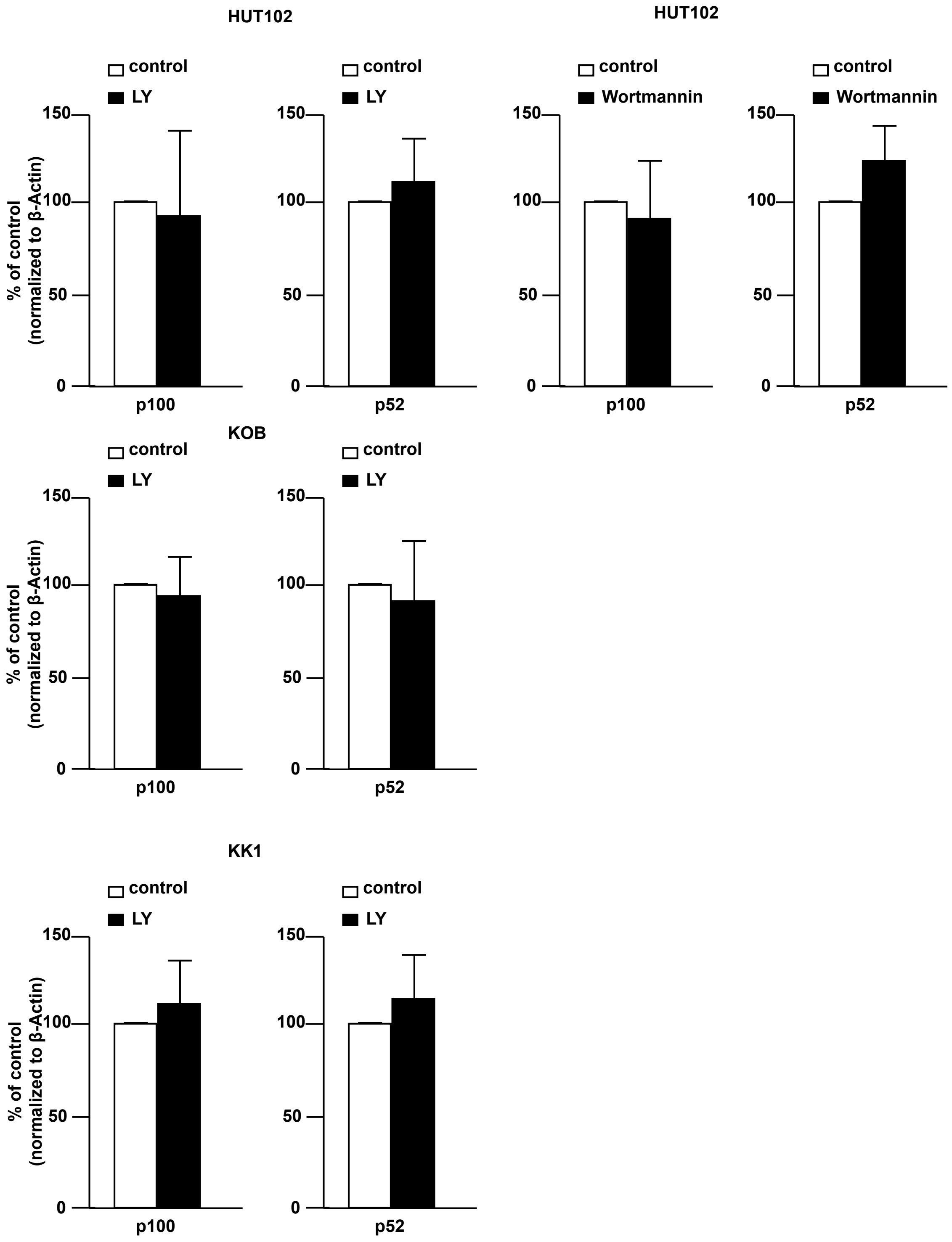

HUT102
HUT102
control
LY
control
LY
control
Wortmannin
control
Wortmannin
% of control
(normalized to β-Actin)
150
100
50
0
p100
p52
p100
p52
KOB
control
LY
control
LY
p100
p52
KK1
control
LY
control
LY
p100
p52

Supplementary Figure 4S. The inhibition of the non-canonical NF-κB signaling pathway in ATL

(a) HUT102 and KOB were treated with increasing concentrations of Okadaic acid (10-1000 nM) and subjected to western blot analysis as indicated. (b) The graph shows relative band intensity of p52. The relative density of the bands is normalized to β-Actin. (c) The graphs show the quantification of relative band intensity of the Okadaic acid (OA)-treated NF-κB signaling pathway in ATL cell lines. The relative density of the bands is normalized to β-Actin. The data are expressed as the mean ± s.d. Student's t-test was used for statistical analysis ($p<0.05$). n=3. (d) T-ALL and ATL cell lines were pretreated with or without MG132 (5 μM) and incubated with 10% FBS DMEM for another 24 h, followed by western blot analysis of NIK and phosphor-NIK (Thr559). Results are representative of three independent experiments. (e) T-ALL and ATL cell lines were subjected to western blot analysis of p100/p52. (f) The graphs show the quantification of relative band intensity of the MG132-treated phosphorylated NIK (Thr559) in ATL cell lines. The relative density of the bands is normalized to β-Actin. The data are expressed as the mean ± s.d. Student's t-test was used for statistical analysis ($p<0.05$). n=3. (g) The graphs show the quantification of relative band intensity of the MG132 and OA-treated phosphorylated NIK (Thr559) in ATL cell lines. The relative density of the bands is normalized to β-Actin. The data are expressed as the mean ± s.d. Student's t-test was used for statistical analysis ($p<0.05$).

a

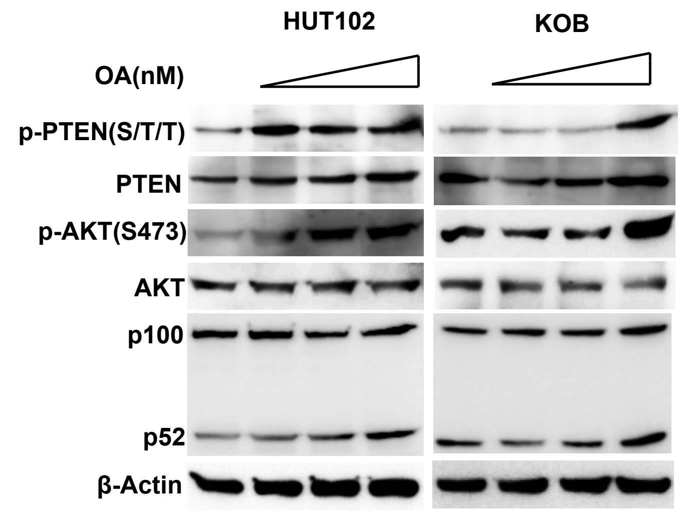
HUT102
KOB
OA(nM)
p-PTEN(S/T/T)
PTEN
p-AKT(S473)
AKT
p100
p52
β-Actin

b

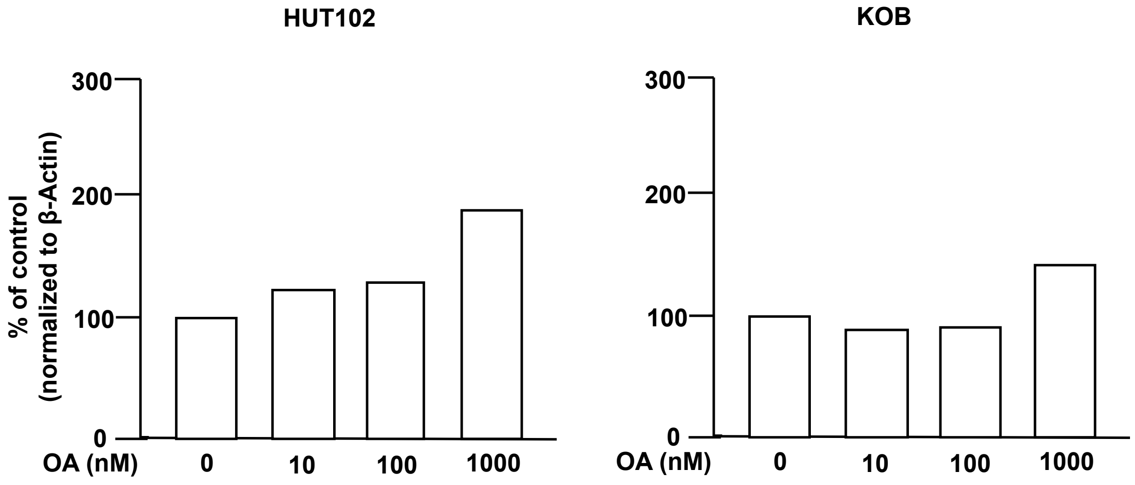
HUT102
KOB
% of control
(normalized to β-Actin)
300
200
100
0
OA (nM)
0
10
100
1000

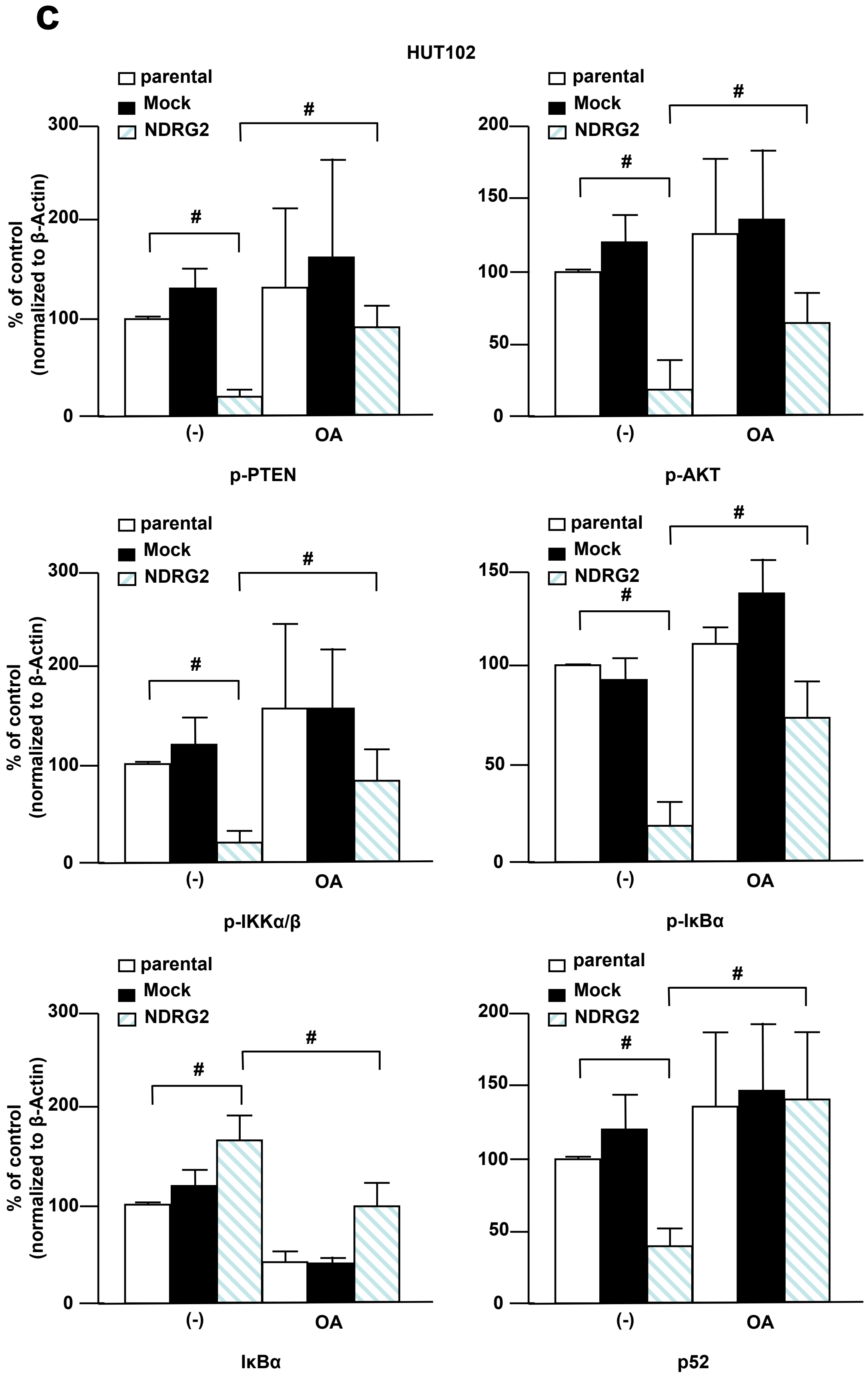
c
HUT102
parental
Mock
NDRG2
% of control
(normalized to β-Actin)
300
200
100
0
150
50
#
(-)
OA
p-PTEN
p-AKT
p-IKKα/β
p-IκBα
IκBα
p52

C

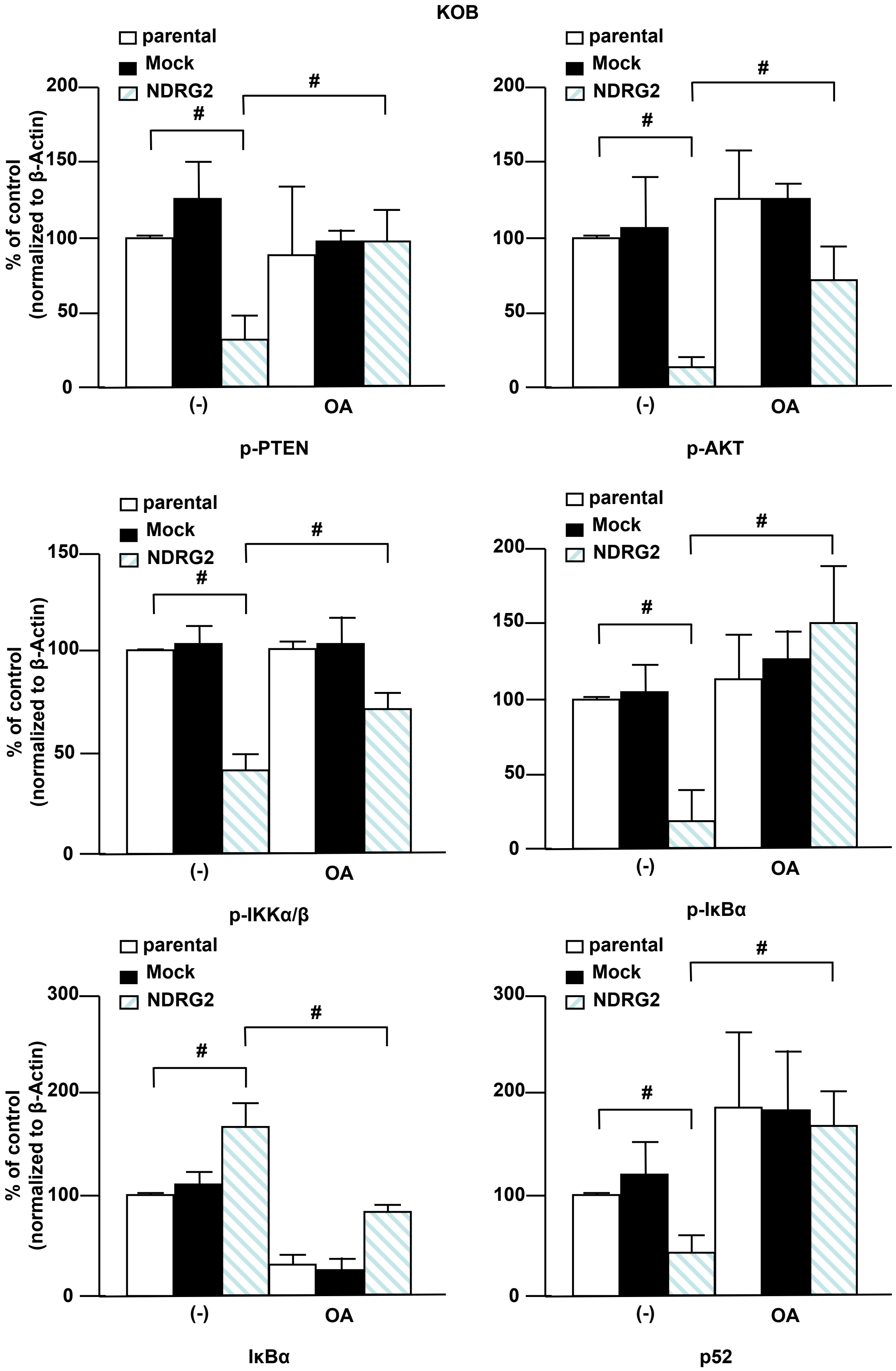
KOB
parental
Mock
NDRG2
% of control
(normalized to β-Actin)
200
150
100
50
0
#
#
(-)
OA
p-PTEN
p-AKT
150
p-IKKα/β
p-IκBα
300
IκBα
p52

d

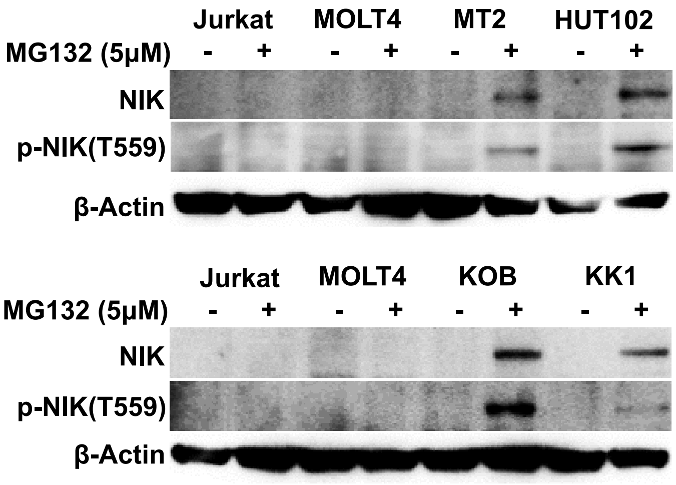
Jurkat
MOLT4
MT2
HUT102
MG132 (5μM)
- + - + - + - +
NIK
p-NIK(T559)
β-Actin
Jurkat
MOLT4
KOB
KK1
MG132 (5μM)
- + - + - + - +
NIK
p-NIK(T559)
β-Actin

e

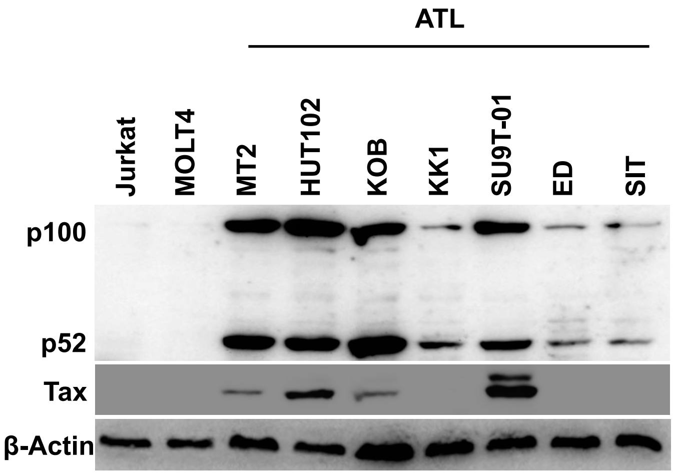
ATL
Jurkat
MOLT4
MT2
HUT102
KOB
KK1
SU9T-01
ED
SIT
p100
p52
Tax
β-Actin

f

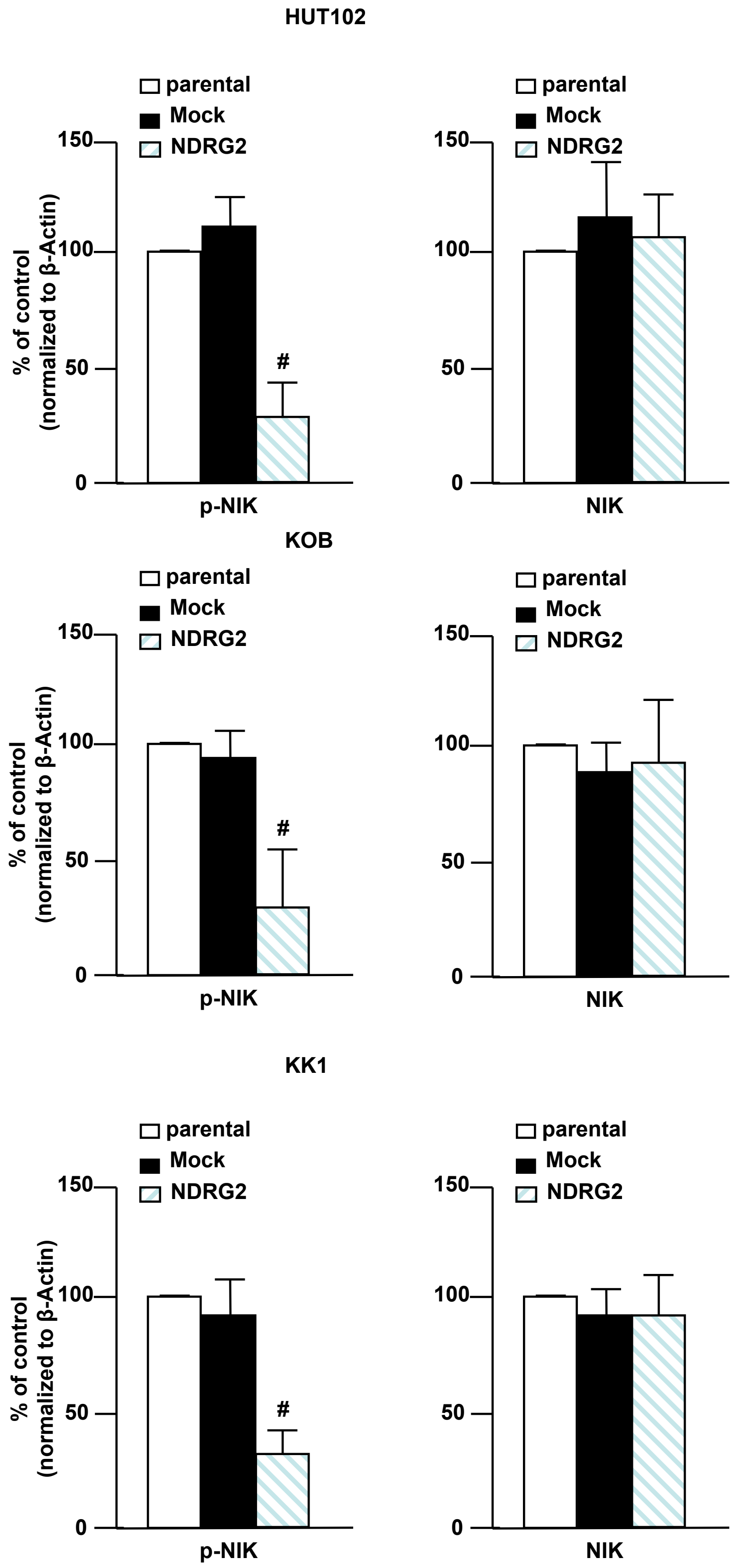
HUT102
parental
Mock
NDRG2
% of control
(normalized to β-Actin)
150
100
50
0
#
p-NIK
NIK
KOB
KK1

g

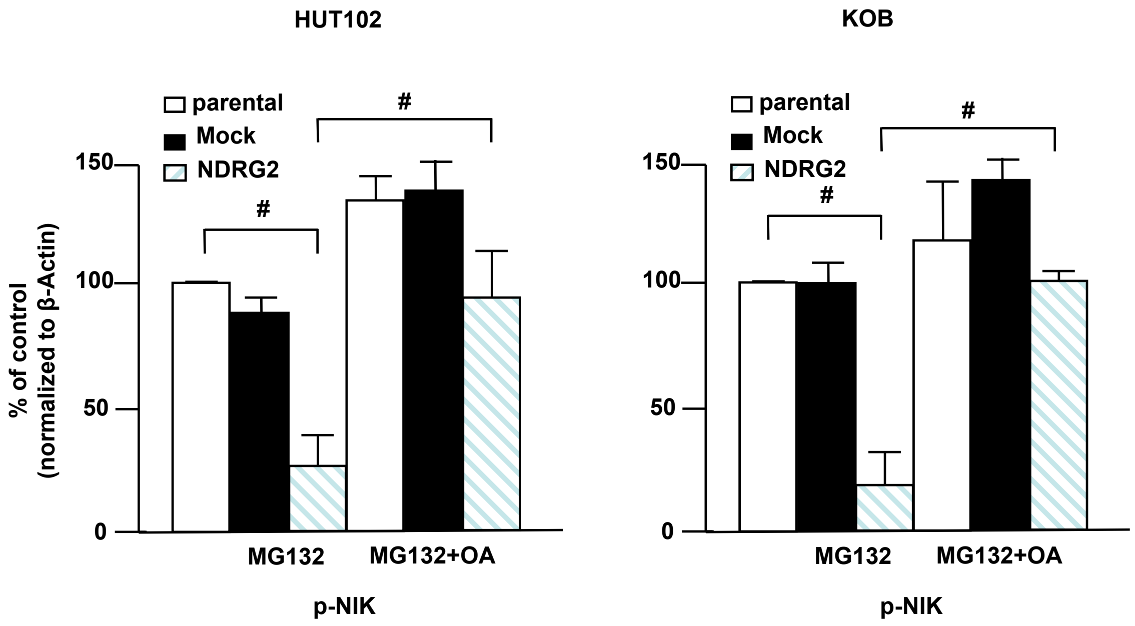
HUT102
KOB
parental
Mock
NDRG2
#
150
100
50
0
% of control
(normalized to β-Actin)
MG132
MG132+OA
p-NIK

Supplementary Figure 5S. Scheme of the NF-κB signaling pathway in control cells (a) and ATL cells (b)

a

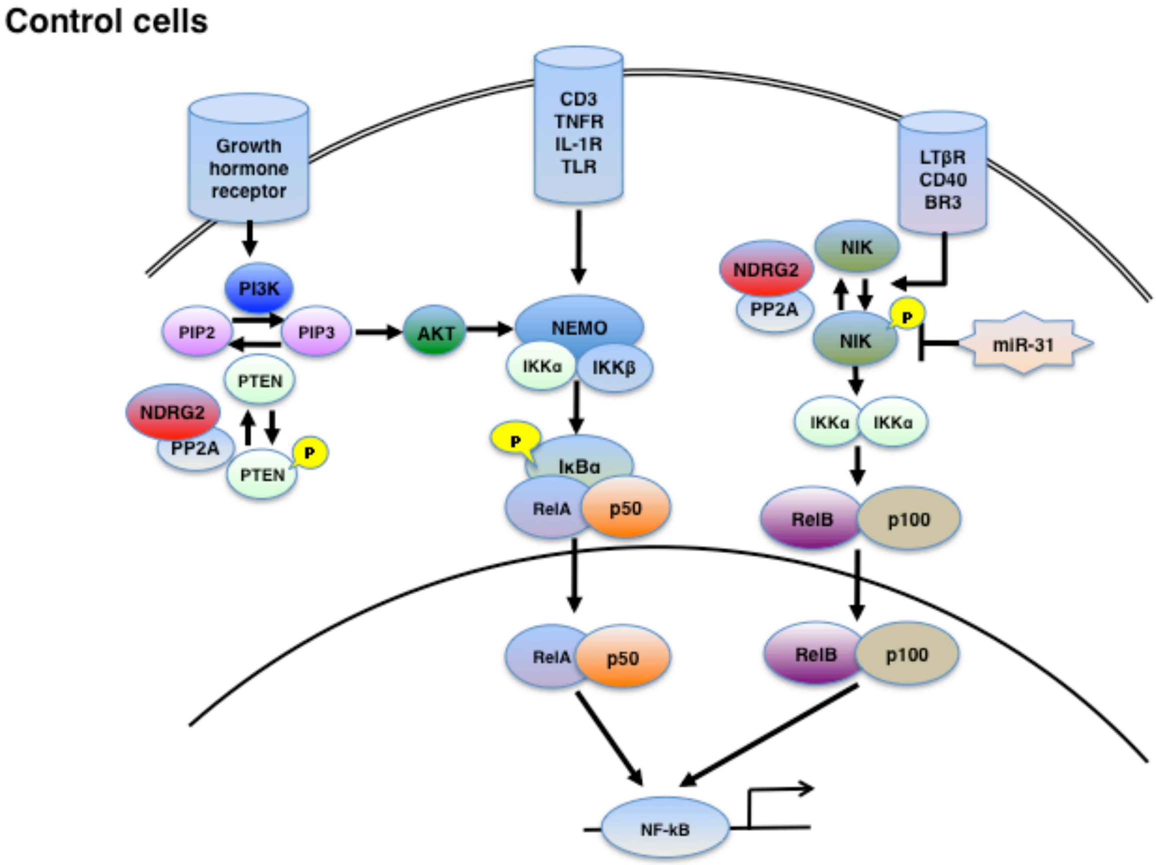

b

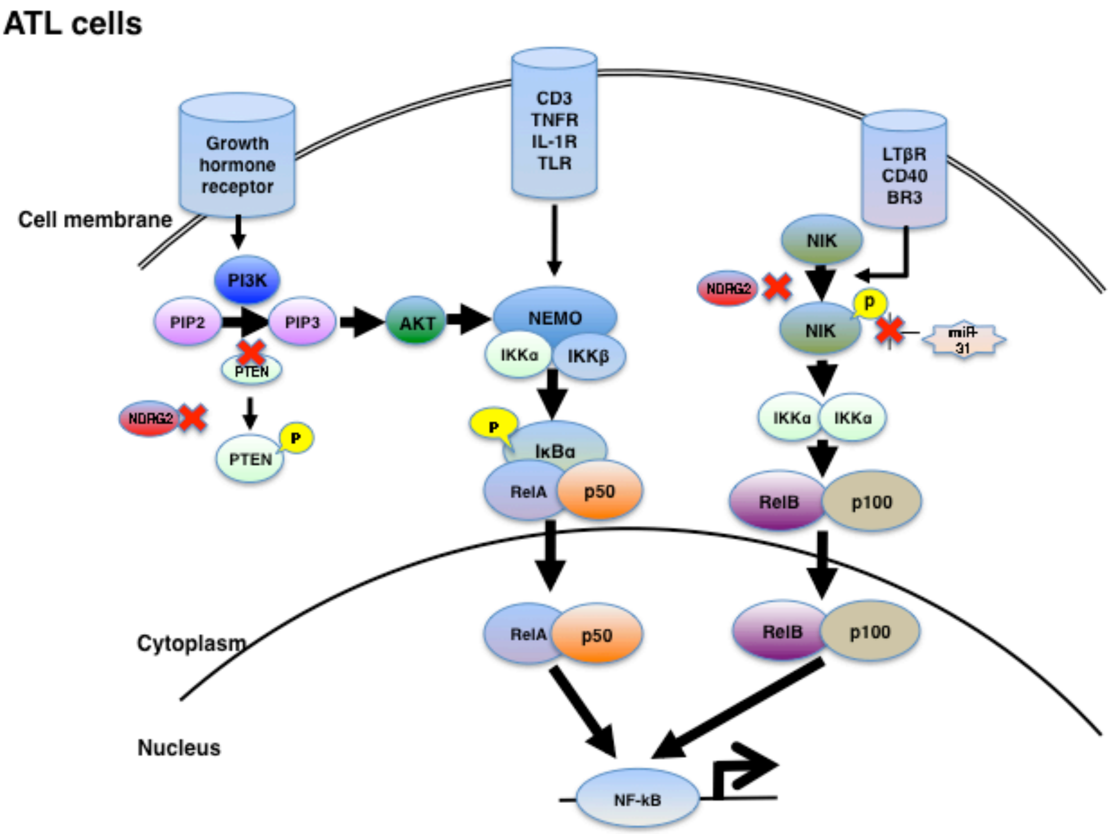
ATL cells
Growth hormone receptor
CD3
TNFR
IL-1R
TLR
LTβR
CD40
BR3
Cell membrane
PI3K
PIP2
PIP3
PTEN
NDRG2
P
AKT
NEMO
IKKα
IKKβ
IκBα
RelA
p50
NIK
miR-31
RelB
p100
Cytoplasm
Nucleus
NF-kB
